# Deep metagenome and metatranscriptome analyses of microbial communities affiliated with an industrial biogas fermenter, a cow rumen, and elephant feces reveal major differences in carbohydrate hydrolysis strategies

**DOI:** 10.1186/s13068-016-0534-x

**Published:** 2016-06-07

**Authors:** Simon Güllert, Martin A. Fischer, Dmitrij Turaev, Britta Noebauer, Nele Ilmberger, Bernd Wemheuer, Malik Alawi, Thomas Rattei, Rolf Daniel, Ruth A. Schmitz, Adam Grundhoff, Wolfgang R. Streit

**Affiliations:** Department of Microbiology and Biotechnology, Biocenter Klein Flottbek, University of Hamburg, Ohnhorststr. 18, 22609 Hamburg, Germany; Institute for General Microbiology, Christian Albrecht University Kiel, Kiel, Germany; CUBE-Division for Computational Systems Biology, Dept. of Microbiology and Ecosystem Science, University of Vienna, Althanstr. 14, Vienna, Austria; Institute of Microbiology and Genetics, Georg-August-University Göttingen, Grisebachstr. 8, Göttingen, Germany; Bioinformatics Core, University Medical Center Hamburg-Eppendorf, Martinistr. 52, Hamburg, Germany; Heinrich Pette Institute, Leibniz Institute for Experimental Virology, Martinistr. 52, Hamburg, Germany

**Keywords:** Anaerobic digestion, Biogas, Biofuels, Biorefinery, Lignocellulosic biomass, Metagenomics, Cellulases, Microbial communities, PULs

## Abstract

**Background:**

The diverse microbial communities in agricultural biogas fermenters are assumed to be well adapted for the anaerobic transformation of plant biomass to methane. Compared to natural systems, biogas reactors are limited in their hydrolytic potential. The reasons for this are not understood.

**Results:**

In this paper, we show that a typical industrial biogas reactor fed with maize silage, cow manure, and chicken manure has relatively lower hydrolysis rates compared to feces samples from herbivores. We provide evidence that on average, 2.5 genes encoding cellulolytic GHs/Mbp were identified in the biogas fermenter compared to 3.8 in the elephant feces and 3.2 in the cow rumen data sets. The ratio of genes coding for cellulolytic GH enzymes affiliated with the *Firmicutes* versus the *Bacteroidetes* was 2.8:1 in the biogas fermenter compared to 1:1 in the elephant feces and 1.4:1 in the cow rumen sample. Furthermore, RNA-Seq data indicated that highly transcribed cellulases in the biogas fermenter were four times more often affiliated with the *Firmicutes* compared to the *Bacteroidetes*, while an equal distribution of these enzymes was observed in the elephant feces sample.

**Conclusions:**

Our data indicate that a relatively lower abundance of bacteria affiliated with the phylum of *Bacteroidetes* and, to some extent, *Fibrobacteres* is associated with a decreased richness of predicted lignocellulolytic enzymes in biogas fermenters. This difference can be attributed to a partial lack of genes coding for cellulolytic GH enzymes derived from bacteria which are affiliated with the *Fibrobacteres* and, especially, the *Bacteroidetes*. The partial deficiency of these genes implies a potentially important limitation in the biogas fermenter with regard to the initial hydrolysis of biomass. Based on these findings, we speculate that increasing the members of *Bacteroidetes* and *Fibrobacteres* in biogas fermenters will most likely result in an increased hydrolytic performance.

**Electronic supplementary material:**

The online version of this article (doi:10.1186/s13068-016-0534-x) contains supplementary material, which is available to authorized users.

## Background

In the context of climate change, the production of biogas as a renewable energy form has become increasingly attractive over the last two decades. Biogas is composed of mainly methane and carbon dioxide, which are produced in a complex and anaerobic microbial process [1]. While the main microorganisms and mechanisms involved in the methane production are well known, the overall process of the microbial biogas producing communities, starting with the anaerobic digestion of the biomass to the final end product, is not well understood [2–5]. Previously, it has been reported that the anaerobic degradation of the plant biomass and the subsequent generation of biogas require the close interaction of many different and phylogenetically diverse microorganisms. Published research implies that the diversity ranges from several hundred to several thousand microbial species in active biogas reactors [3, 4, 6, 7]. Interestingly, it was further reported that the overall production of biogas is probably limited due to the relatively slow hydrolysis of the agricultural plant biomass [8]. Thereby, *Clostridia* appear to play a major role during the initial biomass degradation. In fact, they are the dominant class of hydrolytic organisms in the biogas fermenters [8]. Numerous cellulolytic *Clostridia* produce cellulosomes. Cellulosomes are large multi exoenzyme complexes, whose purpose is the efficient degradation of cellulose [9, 10]. These membrane-associated complexes can be visualized using electron microscopy [11]. While the clostridial systems are, perhaps, the most competitive group within biogas fermenters, they are less dominant in natural digestive organs, such as the cow rumen or the gut of other studied herbivorous animals. Within this context, recent research has uncovered that the *Bacteroidetes* are present in virtually all rumen, gut, and fecal samples of herbivores. Here, they usually represent the predominant bacterial group, besides the *Firmicutes* [12–19]. In contrast to clostridial organisms, bacteria of the phylum *Bacteroidetes* do not produce cellulosomes. However, they are associated with the production of very versatile polysaccharide utilization loci (PULs). PULs are prevalent in the phylum of *Bacteroidetes* and have only recently attracted increasing attention. Evidence is mounting that PULs might play an important part in the breakdown of cellulose [20]. Furthermore, it was recently proposed that cellulolytic PULs might be considered as an alternative system for the degradation of cellulose, next to cellulosomes and free-enzymes [21]. PULs, which were originally described as starch degradation operons, have been predicted in up to 67 *Bacteroidetes* genomes until now. They can be described as a set of genes organized around an *SusC* and *SusD* gene pair [22]. Intrigued by the differences between the composition of the microbiomes of natural cellulolytic systems and biogas fermenters, we wanted to investigate how these differences may affect the ability to effectively degrade biomass in biogas plants. In this study, we employed deep metagenome sequencing in combination with RNA-Seq to obtain detailed insights into the glycoside hydrolase enzymes (GHs), mainly employed in carbohydrate hydrolysis in different cellulolytic systems. Within this paper, we provide evidence that in published natural cellulolytic systems of herbivorous animals the ratio of the *Firmicutes* vs. *Bacteroidetes* is almost 1:1 [14–18, 23, 24]. In contrast, in a technical system, such as biogas fermenters the *Firmicutes* outcompete the *Bacteroidetes* by four-to-six-fold [2–4, 25]. In line with this observation, we show that the overall abundance of potential glycoside hydrolase genes is lower in the biogas fermenter compared to two natural systems due to an underrepresentation of typical rumen and gut bacteria. Furthermore, we wanted to know, if these differences are associated with the predominant transcription of certain GH families, possibly allowing a more efficient degradation of the plant biomass.

## Methods

### Total DNA extraction from an agricultural biogas fermenter sample

Samples were taken from the fermenter of an agricultural biogas plant located near Cologne (Germany) in March and May 2013. At the time of sampling, the biogas plant was running under steady conditions. It produced 536 kW output and was fed with maize silage (69 %), cow manure (19 %), and chicken manure (12 %). Fermentation took place at 40 °C and a pH value of 8 in a 2800 m^3^ fermenter. Total DNA was isolated (Isolation 1) using the QIAamp DNA Stool kit from Qiagen (Hilden, Germany) according to the manufacturer’s protocol for pathogen detection. For this isolation, 2 g of fermenter material were used and the reaction steps were scaled up accordingly. Heating of the suspension was carried out at 95 °C.

For metagenome sequencing, an additional DNA isolation (Isolation 2) from the May sample was conducted using a CTAB-based method according to Weiland–Bräuer [26]. 1.5 g of sample material was mechanically disrupted using a Dismembrator U instrument (Sartorius AG, Göttingen, Germany). Subsequently, 2.7 ml DNA extraction buffer with 5 % CTAB was added to 1 g of homogenized material. Extracted DNA was highly contaminated by humic acids indicated by brownish to yellow color. Contamination was removed using the FastDNA™ SPIN Kit for Soil (MP Biomedicals, Solon, Ohio, US), excluding the initial lysis steps. Purity of DNA was analyzed using a Nanodrop ND-2000 instrument (PEQLAB Biotechnologie GmbH, Erlangen, Germany).

### Amplification and sequencing of 16S rRNA genes

Variable regions of bacterial 16SrRNA genes were amplified as previously published [15] with minor modifications. The V3–V5 region was amplified using the primer set: V3 for 5′-CCATCTCATCCCTGCGTGTCTCCGACTCAGACGCTCGACACCTACGGGNGGCWGCAG-3′ and V5rev 5′-CCTATCCCCTGTGTGCCTTGGCAGTCTCAGCCGTCAATTCMTTTRAGTTT-3′. The primers contained Roche 454 pyrosequencing adaptors, keys, and one unique MID per sample (underlined). To target archaeal 16SrRNA genes, the V4–V6 region was amplified using the primer set: A519F 5′-CCATCTCATCCCTGCGTGTCTCCGACTCAGATATCGCGAGCAGCMGCCGCGGTAA-3′ and A1041R 5′-CCTATCCCCTGTGTGCCTTGGCAGTCTCAGGGCCATGCACCWCCTCTC-3′. The PCR reaction (50 µl) contained 0.5 U of Phusion High-Fidelity DNA Polymerase (Thermo Scientific, Braunschweig, Germany), 10 µl 5× Phusion GC Buffer, 200 µM of each dNTP, 2.5 % DMSO, 1.5 mM MgCl2, 4 µM of each primer, and 20 ng isolated DNA. PCR cycling conditions were: initial denaturation at 98 °C for 3 min, followed by 28 cycles of denaturation at 98 °C for 30 s, annealing at 61 °C for 30 s (archaeal primer set: 66 °C), and extension at 72 °C for 25 s. The final extension was conducted at 72 °C for 5 min. Negative controls were performed with H_2_O instead of template DNA. The obtained PCR products were purified via Gel/PCR DNA Fragments Extraction Kit (Geneaid Biotech, Taiwan) as recommended by the manufacturer. Three separate PCR reactions were conducted for each sample. After gel extraction, the reaction products were pooled in equal amounts. The 16S rRNA gene sequencing was performed at the Göttingen Genomics Laboratory using a Roche GS FLX++ 454 pyrosequencer with titanium chemistry (Roche, Branford, USA).

### Processing and analysis of 16S rRNA genes

Pyrosequencing derived raw sequences were processed according to Wemheuer et al. [27], with the following modifications: After raw data extraction, reads shorter than 300 bp and those possessing long homopolymer stretches (≥8 bp) or primer mismatches (>3 bp) were removed. The sequences were denoised employing Acacia version 1.53b [28]. Chimeric sequences were, subsequently, removed using UCHIME in de novo and in reference mode using the SILVA SSU database (SSURef 119 NR) as reference data set [29, 30]. All non-bacterial as well as singletons OTUs (OTUs containing only one sequence) were removed according to Schneider et al. [31]. The remaining 16S rRNA gene sequences were uploaded to the SILVA NGS (SILVA Next-Generation Sequencing) server for taxonomic classification [29]. Microbial taxonomy was determined using SILVA version 119 and default settings with one adjustment: The cluster sequence identity threshold was increased to 0.99. Rarefaction curves, diversity indices, and shared OTUs were calculated employing the QIIME 1.8 software package [32].

### Metagenome sequencing and de novo assembly

20 ng DNA was sheared with the Bioruptor^®^ (Diagenode; 7 times for 15 s on/90 s off) and libraries were generated using the NEBNext^®^ Ultra™ DNA Library Prep Kit for Illumina^®^ as recommended by the manufacturer. Size and quality of the libraries were assessed using a BioAnalyzer High Sensitivity Chip. Diluted libraries (2 nM) were multiplex-sequenced on the Illumina HiSeq 2500 instrument. Initially, one lane was sequenced for each DNA isolation in paired end mode (2 × 101 bases). Subsequently, a second lane was sequenced for DNA isolation 2 using the same conditions. The number of generated reads is indicated in Table 1. Sequencing was carried out at the Heinrich-Pette-Institut in Hamburg, Germany. Sequencing adapters were removed using Trimmomatic 0.33 [33]. Different de novo assemblies were performed using either the IDBA-UD 1.1.1 [34] or Ray Meta v.2.3.1 [35] assembler (Table 1).

**Table 1:** Number of generated reads and statistics of different de novo assemblies

Biogas fermenter May 2013 sample	#Reads used for assembly	Ray assembly					IDBA-UD assembly				
		#Contigs total	#Contigs >1000 bp	N50 for contigs >1000 bp	Mb in contigs >1000 bp	Mb total	#Contigs total	#Contigs >1000 bp	N50 for contigs >1000 bp	Mb in contigs >1000 bp	Mb total
DNA isolation 1	159,458,382	1,201,371	57,009	7183	209.7	486.0	556,160	116,123	5587	404.7	613.8
DNA isolation 2	421,986,642	2,003,618	94,702	11,536	425.9	876.6	947,772	200,495	5976	724.0	1069.9
DNA isolation 1 + 2	581,445,024	2,319,807	112,571	10,871	512	1,035.8	1,142,608	236,489	5823	843.0	1255.4^a^
DNA isolation 2 + additional sequencing of isolation 2	737,631,618	2,593,366	123,435	12,418	581.3	1161.7^b^					
DNA isolation 1 + 2 + additional sequencing of isolation 2	897,090,000	2,826,937	140,535	11,784	653.73	1292.2					

^a^ Assembly uploaded to IMG/MER and used for the phylogenetic and comparative analyses

^b^ Assembly used for metagenomic binning

### Binning of metagenomic contigs

For metagenomic binning, the assembly was performed using the Ray Meta assembler with a k-mer length of 31. Contig coverage was determined by mapping the initial reads to the contigs using the short-read mapper BBMap (BBMap, Bushnell B., sourceforge.net/projects/bbmap/). Subsequently, Samtools [36] was used to convert, sort, and merge the sam files. After that, BEDTools [37] were used to calculate contig-wise average coverage. As low-coverage and short contigs are known to be error-prone [38], contigs with a length <1 kb and average coverage <3 were discarded from the assembly.

Taxonomic profiling of reads was performed by a sequence similarity search using blastx (NCBI-BLAST 2.2.26, e-value <0.1) [39] against a database of universally conserved proteins which occur in 98 % of all eukaryotes, bacteria, and archaea. The database was clustered to a sequence similarity level of 97 % to remove redundancy. Blast results were taxonomically assigned by MEGAN [40] with min. bitscore 60 and min. support percent 0.05, and visualized by Krona 2.5 [41].

Taxonomic profiling of contigs was performed using AMPHORA2 [42] using the universal marker set of 31 genes. NCBI taxonomy ids were mapped to phylogenetic lineages given by AMPHORA2. The comparison between read-based and assembly-based communities was made to verify the consistency between the sample and the assembly. The visualization software Elviz [43] was used to visualize contig coverages, length, GC content, taxonomy, and to assess possible binning strategies. For binning based on composition and differential coverage data, the software CONCOCT was used with default parameters [44]. As suggested in the CONCOCT documentation, contigs were cut up into sequences of 10 kb length. Subsequently, mapping of the initial reads was carried out using Bowtie2 [45] to determine the coverage of these contigs. Checkm 1.0.3 [46] was used to assess the completeness and contamination of the bins. Strongly contaminated bins were inspected by VizBin [47], which allowed a further separation of bins, if they formed two or more distinct clusters. Genes encoding presumable carbohydrate-active enzymes were annotated based on sequence similarity to sequences in the CAZY database [48]. The CAZy database (May 2015) was downloaded using a custom Python script. After this, a blastp sequence similarity search [49] of open reading frames, which were extracted from the assembly using getorf [50], was performed against the CAZy database using default parameters and an e-value cutoff of 1e–20.

All bins with completeness >80 %, contamination <10 % and heterogeneity (of the contamination) <50 % were classified as “high quality”. Contigs belonging to these bins were removed from the assembly. According to the CONCOCT workflow, the remaining contigs were binned again using CONCOCT. The resulting bins were again evaluated using Checkm. The taxonomy of the bins was obtained from the AMPHORA2 results by determining the consensus lineage of all bin-specific marker genes (cut-off confidence scores >0.8). Annotation of the genome bins was performed using the annotation framework ConsPred V1.21 (http://sourceforge.net/p/conspred/wiki/Home/). In the file “conspred_input_specification.txt”, the parameters “taxon exclude”, “minimal number rRNA”, and “minimal number tRNA” were set to “0”.

### Identification of carbohydrate-active gene candidates, PULs, and cellulosomal scaffoldin proteins

The assembled metagenomic contigs (biogas and elephant) and scaffolds (cow) were subjected to gene prediction using Prodigal 2.6.1 in meta mode [51]. The number of predicted open reading frames (ORFs) for the respective metagenome is indicated in Table 4. Amino acid sequences of the predicted ORFs were screened for similarity to glycoside hydrolase (GH) families and carbohydrate esterase (CE) families as classified in the CAZy database [48]. For this screening, profile hidden Markov models (HMMs) based on the respective CAZy families were downloaded from the dbCAN database [52] and compared to the protein sequences using hmmscan of the HMMER 3.1b1 software package (hmmer.org). All resulting hits were processed as recommended by the author of the dbCAN database. First, overlapping hits were removed; the hit with the higher e-value was discarded. Hits not covering at least 30 % of the respective HMM were also removed. For the remaining hits, an e-value cutoff of 1e–5 for alignments longer than 80aa and 1e–3 for alignments shorter than 80aa was applied. For the GH109 family, a custom made model was used and the covered fraction of the HMM was increased to 55 %. Duplicate hits in the family GH74 were removed by hand. To identify potential bacteroidetal PULs, the dbCAN database was extended by two additional models: a model for SusD like proteins (PF07980) downloaded from the Pfam database (http://www.pfam.xfam.org/) and a model for TonB-dependent receptor/SusC like proteins (TIGR04056) downloaded from the TIGRfam database (http://www.tigr.org/TIGRFAMs). To identify potential cellulosomal gene clusters in the respective metagenomic data set, we used amino acid sequences of known cellulosomal scaffolding proteins for an iterative protein sequence similarity search via Jackhmmer (hmmer 3.1 package). For this search, a score cut-off value of 700 was applied, and the following scaffoldin query sequences were used (NCBI accession numbers and organism names in brackets): cbpA (AAA23218.1, *Clostridium cellulovorans*), CipC (AAC28899.2, *Clostridium cellulolyticum H10*), cipA (BAA32429.1, *Clostridium josui*), cipA (AAK78886.1, *Clostridium acetobutylicum ATCC 824*), cipA (Q06851, *Clostridium thermocellum*), ScaA (AAG01230.2, *Pseudobacteroides cellulosolvens*), ScaB (AAT79550.1 *Bacteroides cellulosolvens*), and ScaB (CAC34385.1, *Ruminococcus flavefaciens 17*). To allow a comparison between the different sized assembled metagenomic data sets, the number of potential GH and CE gene hits was normalized to 1 Gb of assembled DNA for all comparative analysis.

### Taxonomic assignment of carbohydrate-active gene candidates

Amino acid sequences of ORFs, which were previously assigned to GH families associated with cellulases and CE families, were used for a protein blast search against the NCBI non-redundant database. The number of maximal target sequences was decreased to 20 and an e-value cutoff of 1e–2 was employed for this search. Next, all obtained hits were loaded into MEGAN5 [40] and the lowest common ancestor (LCA) algorithm (default settings, unless otherwise specified) was used to classify the sequences taxonomically.

### RNA extraction and sequencing from an elephant feces sample

A feces sample from an adult female zoo elephant was taken in April 2014 in the same way and from the same animal described in the publication by Ilmberger et al. [15] living in the Hagenbeck Zoo in Hamburg, Germany. The sample was transported to the lab on ice and then stored at −70 °C. Isolation of ribonucleic acids for RNA-Seq was carried out using the PowerMicrobiome™ RNA Isolation Kit from Mo Bio Laboratories (Carlsbad, Germany) as recommended by the manufacturer. In a next step, ribosomal RNA was depleted using the Ribo-Zero™ rRNA Removal Kit for Bacteria (Illumina, Madison, USA) according to the manufacturer’s instructions. The remaining transcripts were fragmented and cDNA libraries for Illumina sequencing were constructed by Vertis Biotechnology AG, Germany (http://www.vertis-biotech.com/), as described previously for eukaryotic microRNAs [53], but omitting the RNA size-fractionation step prior to cDNA synthesis. Equal amounts of RNA samples were poly(A)-tailed using poly(A) polymerase. Then, the 5′-triphosphates were removed by applying tobacco acid pyrophosphatase (TAP) resulting in 5′-monophosphat. Afterwards, a RNA adapter was ligated to the 5′-phosphate of the RNA. First-strand cDNA was synthesized by an oligo(dT)-adapter primer and the M-MLV reverse transcriptase. In a PCR-based amplification step, using a high-fidelity DNA polymerase, the cDNA concentration was increased to 20–30 ng/µl. A library-specific barcode for multiplex sequencing was part of a 3′-sequencing TruSeq adapter. The resulting cDNA libraries were sequenced using a HiSeq 2500 machine in single-read mode running 100 cycles.

### RNA extraction and sequencing from a biogas fermenter sample

A biogas fermenter sample was taken in March 2015, mixed with an equal amount of RNAlater solution and immediately frozen on dry ice for transport. In the lab, the sample was stored at −70 °C.

Isolation of ribonucleic acids for RNA-Seq was carried out using the PowerMicrobiome™ RNA Isolation Kit from Mo Bio Laboratories (Carlsbad, Germany), as recommended by the manufacturer. In a next step, ribosomal RNA was depleted using the Ribo-Zero™ rRNA Removal Kit for Bacteria (Illumina, Madison, USA) according to the manufacturer’s instructions. The rRNA-depleted samples were purified via the RNA Clean & Concentrator Columns from Zymo Research (Irvine, USA). During this step, an additional in-column DNase I treatment was included to ensure complete removal of DNA. Subsequently, synthesis of double-stranded cDNA was conducted using the Maxima H Minus Double-Stranded cDNA Synthesis Kit from ThermoScientific (Waltham, USA). In the first-strand cDNA synthesis reaction, 2 µl of random hexamer primer were used. Final purification of the blunt-end double-stranded cDNA was carried out using SureClean Plus solution from Bioline (Luckenwalde, Germany). The cDNA was sequenced in the same way as the total DNA. To achieve the required amount of cDNA for library preparation, multiple RNA isolations from the same sample were pooled.

### Processing and analysis of RNA-Seq reads

To identify highly transcribed glycoside hydrolases in the biogas fermenter and elephant feces samples, RNA-Seq reads generated for both samples were checked for read quality and sequencing adapters were removed using Trimmomatic 0.33 [33]. In the next step, poly(A) tails >10 were removed using the trim_tail_left/right function of PRINSEQ lite 0.20.4 [54]. Subsequently, short sequences (≤99 nt) were filtered and rRNA gene-derived sequences were removed employing SortMeRNA 2.0 [55]. All remaining non-rRNA reads were used for mapping to the metagenomic contigs of the respective assembly (indicated in Table 4). For this mapping, Bowtie2 [45] was used in end-to-end mode (preset: very sensitive) and allowing 1 mismatch during seed alignment. After this, the htseq-count script from HTSeq 0.6.1 [56] was applied in non-stranded mode with default settings (alignment quality cutoff <10) to count the reads which map to genes predicted in the respective assembly by Prodigal 2.6.1. Finally, the genes were filtered for previously identified potential glycoside hydrolase encoding genes. The taxonomic origin of 100 GHs (including all CAZy families) and 50 GHs (including only cellulolytic CAZy families) with the highest numbers of mapped cDNA reads was determined via a protein blast and MEGAN5 LCA analysis as described above.

To analyze the expression level of cellulolytic GHs genes in the bacterial metagenomic bins created from the biogas fermenter metagenome, the initial raw reads were again processed using PRINSEQ lite: 10 bases were trimmed from the 5′ end, bases with a quality score <5 were trimmed from the 3′ end, and sequences with mean quality <20 or length <70 bp were discarded. Subsequently, the remaining RNA-Seq reads were mapped to the Ray assembly used for metagenomic binning (indicated in Table 1) via Bowtie2. Next, Bedtools multicov [37] was used to calculate coverage values of potential CAZy glycoside hydrolases which were identified in the metagenomic bins as described in the binning section. The coverage values were converted to rpkm values and plotted against bin taxonomy using the heatmap.2 function of the gplots package in R (R Core Team, 2015).

### Transmission electron microscopy (TEM)

Slices were prepared using the microtome Reichert-Jung Ultracut E. Fixation was performed in 2 % glutaraldehyde in 75 mM cacodylate buffer (pH 7.0). Next, the samples were supplied with 2 % agar in 75 mM cacodylate buffer (pH 7.0) and further fixed with 1 % OsO_4_ in 50 mM cacodylate buffer (pH 7.0). After washing with 75 mM cacodylate buffer (pH 7.0), water was removed with acetone and the sample was infiltrated with Spurr resin (Polysciences, Warrington, PA, USA). TEM pictures were taken on the LEO 906 E using the camera Gatan 794 and the software Digital micrograph (Gatan GmbH, Munich, Germany).

### DNS Assay for determination of total cellulolytic activities

To determine total cellulolytic activities in the sample materials, 0.2 g of biogas fermenter material as well as various fecal samples of herbivorous animals were diluted in 1 ml phosphate buffer (0.1 M, pH 6.6) containing 2 mM EDTA and 1 mM PMSF. The feces samples were obtained from animals living in the Hagenbeck Zoo in Hamburg, Germany. Subsequently, the samples were sonicated on ice for 15 min and centrifuged for 1 min at full speed. The supernatants were transferred to new tubes and 100 µl aliquots were used for total protein quantification via the Pierce™ BCA Protein Assay Kit as recommended by the manufacturer (Thermo Fischer Scientific, Pinneberg, Germany). In the next step, 100 µl of the remaining supernatants were used for the 3,5-dinitrosalicylic acid (DNS) assay. The assay was conducted in triplicates as described by Juergensen and colleagues [57]. Incubation of the samples with carboxymethycellulose (CMC) was carried out at 37 °C for 90 min. For each sample, an additional reaction with buffer instead of CMC was conducted and used as a blank. The amount of reducing sugar ends was quantified at 546 nm using a SmartSpec Plus spectrophotometer (Biorad, Munich, Germany). For the calculation of specific enzyme activities, the obtained values were corrected against the measured total protein content. Data are mean values of three independent tests. One unit is defined as the amount of enzyme generating 1 µmol of reduced sugar per minute.

### Sequence data deposition

Within the framework of this study, generated raw sequence data have been deposited under the NCBI BioProject number PRJNA301928. In addition, an assembly of the biogas fermenter metagenome can be accessed and downloaded via IMG/ER (https://www.img.jgi.doe.gov) using the IMG ID 3300002898. The 104 metagenomic bins are provided in the compressed Additional files 1, 2, 3 and 4.

## Results and discussion

### Characteristics of the analyzed biogas plant

Biogas plants harbor complex microbial communities that are essential for the different steps of biogas production. However, the overall biogas production rates are limited and depend on the initial hydrolysis of the plant biomass [2, 8, 58]. To identify possible limitations regarding the overall hydrolytic performance of biogas plants, we sampled and analyzed a typical one-stage agricultural plant with respect to its taxonomic structure and its metagenome content. A detailed overview about the process parameters of this plant is provided in the “Methods” section, and additional parameters are shown in Additional file 5: Table S1. Given the fermentation and process conditions, this plant is representative for several thousand one-stage plants across Europe. Samples were taken at two time points (March and May 2013) for DNA extraction and one time point (March 2015) for RNA extraction as described in the “Methods” section.

### Community structure and diversity of the agricultural biogas plant

**Fig. 1:**
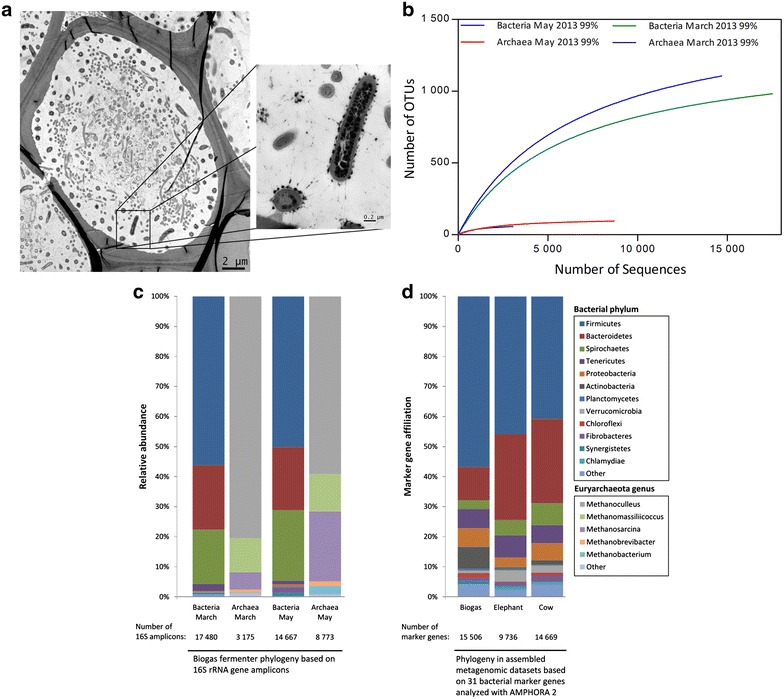
**a** TEM micrograph of a decomposing plant cell and the associated microorganisms in a biogas fermenter. Cellulosome-producing bacteria are almost exclusively observed in close association with the plant cell wall, where they appear to suppress growth of other microbes. Most other microorganisms are located at the more central part of the decomposing plant cell. Cellulosome-producing bacteria were identified by the large *dark spots* attached to the cells. **b** Rarefaction *curves* calculated for two fermenter samples of the studied agricultural biogas plant. The OTUs were clustered at 99 % genetic similarity of 16S rRNA genes. The sequences were denoised employing Acacia, and chimeric sequences were removed using UCHIME. Singleton OTUs were removed prior to the rarefaction analysis. **c** Phylogenetic analysis of two biogas fermenter samples based on 16S rRNA gene amplicons. The *bars* indicate the relative abundance of bacterial phyla and euryarchaeota genera in two samples taken from the same fermenter at different time points (March and May 2013). **d** Phylogenetic analysis of three assembled metagenomic data sets based on 31 bacterial marker genes. The *bars* show the marker gene affiliation to bacterial phyla in the data sets derived from biogas fermenter May sample and for reasons of comparison from published data sets of elephant feces and cow rumen samples [15, 23]. For this analysis, the AMPHORA 2 software was used

To analyze the community structure and the main actors in lignocellulose degradation, transmission electron microscopy (TEM), 16S rRNA gene amplicon, and metagenome sequencing were conducted. As expected, TEM microscopy indicated a high microbial diversity and a substantial number of cellulosome-producing bacteria in the studied biogas sample (Fig. 1a). Surprisingly, image analysis of several large decomposing plant cells implied that the cellulosome-producing microorganisms were in general closely attached or in proximity to the decomposing cell walls, while other microbes were only observed in some distance from the degrading cell walls. This was also observed when other samples from the same biogas reactor were analyzed using TEM (Additional file 6). In fact, the cellulose decomposing bacteria formed a loose layer or biofilm that was mostly not penetrated by other microorganisms. This is an intriguing and novel observation, since it implies that the cellulosome-producing bacteria have a competitive advantage and that they can colonize and degrade the plant material in the absence of other bacteria. A similar observation was not made when samples from elephant feces [15], a herbivore that is known for its richness in cellulolytic enzymes, were analyzed. Intrigued by this observation, we assayed total cellulolytic activities in the supernatant of biogas fermenter content and feces samples obtained from various herbivores. In all cases, the biogas sample had the lowest cellulolytic activities (Additional file 7). It was approximately three–five-folds less active than supernatants obtained from mara, elephant, cow, and zebra feces samples. Even though carboxymethylcellulose is a model substrate which cannot reflect the total hydrolytic activity of diverse glycoside hydrolases and sample treatment (e.g. sonication) might have an effect on the initial activity, this finding suggested that there were major differences within the bacterial communities which would lead to the observed different activity profiles.

To get a further insight into the diverse community of the studied biogas plant, 16S rRNA gene amplicon sequencing was carried out. Clustering of bacterial 16S rRNA gene amplicons at a 99 % similarity level resulted in 994 OTUs for the March 2013 sample and 1108 OTUs for the May 2013 sample (Fig. 1b). Archaeal diversity was substantially lower in the samples. Archaeal-derived 16S rRNA gene amplicons were clustered to 59 (March) and 95 (May) OTUs. An additional information regarding diversity and richness (Chao1, Shannon-Index) is provided in Additional file 5: Table S2. A direct comparison between the samples from our plant indicated that 691 bacterial and 52 archaeal OTUs could be observed in both analyzed samples Additional file 5: Table S3. This relatively high number of shared OTUs suggests the presence of a mostly steady microbial core community in the biogas fermenter under the constant process conditions. Within this framework, the taxonomic classification of the bacterial 16S amplicons indicated that most of the obtained sequences (March 56 %, May 50 %) were affiliated with the phylum of the *Firmicutes* (Fig. 1c). Other abundant phyla were the *Bacteroidetes* with a relative abundance of 21 % in both samples and the *Spirochaetes* (March 18 %, May 24 %). The archaeal-derived 16S rRNA amplicons were all classified into the phylum of *Euryarchaeota.* In both samples, the most abundant archaeal genus appeared to be *Methanoculleus* (March 81 %, May 59 %). The next abundant genera were *Methanomasiliicoccus* (March 11 %, May 13 %) and *Methanosarcina* (March 6 %, May 23 %). A more detailed taxonomic breakdown of the 16S rRNA gene analysis results is given in Additional file 8.

### Metagenome-based analysis and binning of the May 2013 biogas sample

In addition to amplicon sequencing, a large data set of metagenomic DNA was produced for the May sample. We generated 581 million reads which were assembled to 236,489 contigs (>1000 bp) with a total of 2 million potential open reading frames and 1.25 Gb of assembled DNA (Table 1). This data set comprises the largest currently assembled and published data set for a biogas reactor so far and it is, with respect to cellulolytic communities, the second largest metagenome currently published. Only the data set obtained from a microbiome adherent to switchgrass, which was incubated in the rumen of a cow for 72 h, is larger with respect to the assembly [23].

We utilized this comprehensive assembly to further investigate the community structure in the fermenter and found distinct differences in the phylogenetic make up and the overall diversity compared to the amplicon-based data. For this, we employed the AMPHORA2 software [42], which uses 31 conserved bacterial proteins as phylogenetic markers. A total of 15,506 marker genes were identified in our biogas metagenome data set and classified. Thereby, the analysis revealed that 57 % of the marker genes were affiliated with the *Firmicutes* followed by *Bacteroidetes* (11 %), *Actinobacteria* (7 %), *Tenericutes* (6 %), *Proteobacteria* (6 %), *Spirochaetes* (3 %), and other phyla (Fig. 1d). Almost 50 % of all identified marker genes were assigned to the class *Clostridia* followed by *Bacteroidia* (9 %), *Actinobacteria* (7 %), *Mollicutes* (6 %), and other classes.

In general, the phylogenetic structure of our sampled biogas plant appeared to be similar to the structures described in already published studies of biogas fermenters. These published studies were in part based on 16S rRNA amplicon analyses, but also based on metagenome data sets [2–4, 25]. In these studies, it was repeatedly reported that the *Firmicutes* were the prevalent phylum followed by the *Bacteroidetes*. By comparing the ratios of the *Firmicutes* versus the *Bacteroidetes* in already published studies of agricultural biogas fermenters and our own analysis, we found that the mean ratio of the *Firmicutes* versus the *Bacteroidetes* was 5.6–6.0:1 (Table 2 and included references) indicating an, on average, almost six-fold higher relative abundance of the *Firmicutes* compared to the *Bacteroidetes* in the analyzed fermenters.

**Table 2:** Ratio of the phyla *Firmicutes* vs. *Bacteroidetes* in biogas fermenters and herbivorous animals

Microbiome/community	*Firmicutes/Bacteroidetes* phyla ratio		Reference/data source
	16S amplicon-based analysis	Metagenome-based analysis	
Biogas fermenter	2.4:1	5.2:1	This study
Biogas fermenter	–	4.7:1	[25]
Biogas fermenter (dry fermentation)	–	4.1:1	[3]
Biogas fermenter (wet fermentation)	–	3.9:1	[3]
Biogas fermenter	9.6:1	5.9:1	[4]
Biogas fermenter	–	9.6:1	[2]
*Mean*	*6:1*	*5.6:1*	
Asian elephant feces	0.8:1	1.6:1	[15]
Switchgrass incubated in cow rumen	–	1.5:1	[23]
Rumen of a hay-fed cow	0.8–1.7:1	–	[14]
Svalbard reindeer Rumen	0.5:1	0.4:1	[18]
White rhinoceros feces	1.6–2.7:1	–	[24]
Rex rabbit feces	0.8–1.3:1	–	[16]
Rumen of a pasture-fed sheep	0.3–0.5:1	–	[14]
Giraffe rumen	1.6:1	–	[17]
*Mean*	*1:1*	*1.2:1*	

The data are based on the data sets published in the indicated references or on data produced in this study

To further examine the microbial community in the biogas fermenter, a metagenomic binning based on composition and differential coverage data was performed for the May 2013 sample. For this analysis, the Ray Meta assembly with the highest N50 value was used (Table 1) and binning was conducted. Thereby, 104 high-quality bins were observed and assigned to four binning categories (Table 3). The binning results basically reflected the population structure as determined by the marker gene analysis. In total, 57 of the high-quality bins were affiliated with the *Firmicutes* and most of these with the class Clostridia (51). The second most abundant taxonomic bin classification was *Bacteroidetes* with 21 observed bins of which 16 were further attributed to the class *Bacteroidia*. The remaining bins were mainly affiliated with the *Fibrobacteres* (3), the *Spirochaetes* (4), the *Actinobacteria* (2), the *Verrucomicrobia* (2), and the *Euryarchaeota* (3). Nine bins were not assigned to a specific bacterial phylum. A detailed overview about the taxonomic classification of the bins, the estimated bin completeness, the bin contamination, and the size of the bin is provided in Additional file 5: Table S4. For some of the bins, a taxonomic classification with high confidence was possible down to the species level. For example, highly complete genome reconstructions were possible for *Fibrobacter succinogenes* or a closely related species (Bin-IDs pb121 and pb122). In addition, the binning of genome drafts of the cellulolytic bacteria *Clostridium thermocellum* (Bin-ID 96) and *Clostridium phytofermentans* (Bin-IDs pb35-2, pb186-2, and pb35-1) was possible. In the class *Bacteroidia*, comprehensive genome binnings were obtained for species closely related to *Paludibacter propionicigenes* (Bin-IDs 145 and 201) and a not further classified organism of the family *Porphyromonadaceae* (Bin-ID pb69). Finally, a genome bin of the methanogenic archaeon *Methanosarcina barkeri* was reconstructed with only minor contamination (Bin-ID pb85). Besides the known presence of methanogenic archaea within biogas fermenters, the number of high-quality bins related to this taxonomic group was rather small compared to the number of bins obtained for bacteria. In general, the binning of metagenomic contigs into high-quality genome bins allows the reconstruction of key metabolic features of these organisms or OTUs. This is especially useful for, so far, under-represented groups which have not been studied in much detail. Thus, the bins generated during this study provide the basis for a future in-depth analysis of the metabolism and physiology of these organisms.

**Table 3:** Binning summary of biogas fermenter May 2013 sample

Bin category	Quality criteria	# of bins in category
Good bins	>95 % completeness and <5 % contamination or	20
	>95 % completeness and <10 % contamination with >90 % heterogeneity	
Nearly complete genome drafts	>90 % completeness and <5 % contamination	20
Nearly complete pangenome drafts	>90 % completeness and >5 % contamination	37
Incomplete genome drafts	60–90 % completeness and <7 % contamination	27

### A direct comparison of the biogas plant microbial community with fecal and rumen microbiomes of herbivores indicates major differences in the ratio of the *Firmicutes* versus the *Bacteroidetes*

Based on the phylogenetic analysis of the biogas fermenter analyzed in this study and the studies listed in Table 2, we asked if the relative ratio of the phylum of the *Firmicutes* versus the *Bacteroidetes* could be used as an indicator for the fitness of a biogas plant and if this would be indicative for the diversity of plant biomass degrading genes. Since most agricultural biogas fermenters are inoculated and fed with various animal manures, it is likely that the *Bacteroidetes* are present at high levels initially, but are then outcompeted. Reasons for this shift in the microbial community might be the operation conditions in the biogas reactors or the lack of growth factors that are usually present in the natural habitats of the *Bacteroidetes*. Within this framework, it is noteworthy that our fermenter was also fed with cow manure and chicken manure that both contain high levels of the *Bacteroidetes* [59, 60].

In a next step, we calculated the ratios of the *Firmicutes* versus the *Bacteroidetes* in published fecal, rumen, and gut samples of herbivorous animals which nurture from various plant-derived biomasses. The resulting ratios, as well as the methods used in the original publication for the analysis of the microbial community, are indicated in Table 2. Interestingly, when we analyzed the published data, we observed a mean ratio of almost 1:1 (*Firmicutes* vs. *Bacteroidetes*) for seven out of eight analyzed natural microbiomes. Only the published microbiome of the white rhinoceros revealed a slightly higher ratio of 1.6–2.7:1. Altogether, these analyses imply an almost equal abundance of *Firmicutes* and *Bacteroidetes* in these natural systems. This is in contrast to the ratios observed for the biogas fermenter in this study and many other published examples. In all these studied biogas fermenters, the *Firmicutes* were usually 4–6 times more prominent than the *Bacteroidetes*. Thus, it is likely that bacteria affiliated with *Bacteroidetes* do not compete as well in agricultural biogas plants compared to their natural habitats and compared to the *Firmicutes*. It is tempting to speculate that a decreased abundance of the *Firmicutes* together with an increased abundance of the *Bacteroidetes* might be an indicator for the fitness of biogas plants with respect to cellulolytic activities.

### In-depth analysis of predicted glycoside hydrolase and carbohydrate esterase family enzyme abundance and origin largely confirms the phylogenetic analyses and implies a lower enzyme abundance compared to natural microbiomes

Intrigued by the above-made findings, we wanted to investigate how the different ratios of the *Firmicutes* vs. the *Bacteroidetes* might affect the abundance of genes encoding GH family enzymes which are involved in the breakdown of the plant biomass and especially the lignocellulose. To address this question, we analyzed our assembled metagenomic data set obtained from the biogas fermenter with respect to the predicted diversity of hydrolytic enzymes involved in lignocellulose degradation and with respect to the taxonomic origin of these genes and enzymes. For this analysis, we used profile hidden Markov models, which were based on entries in the carbohydrate-active enzyme database (CAZy). The CAZy database encompasses a large set of validated carbohydrate-active enzymes and offers a sequence-based family classification of enzymes that are involved in the modification or breakdown of polysaccharides [48]. Within the up to 2 million-predicted potential genes of the analyzed biogas fermenter sample, we identified a total of 17,305 putative genes for glycoside hydrolases from 109 different families. This equals 13.8 GHs per Mbp of assembled DNA. With respect to the lignocellulose degradation, the most predominant GH families observed were GH3, GH5, GH9, GH51, GH74 and GH94 family enzymes. The total number of potential hits observed in the respective family was 977 for GH3, 599 hits for GH5, 216 for GH9, 265 for GH51, 373 for GH 74 and 269 for GH94. These GH families encompass a variety of hydrolytic enzymes, e.g. cellulases, endo- and exoglucanases, arabinofuranosidases, endoxylanases, cellobiohydrolases, and xyloglucanases.

**Fig. 2:**
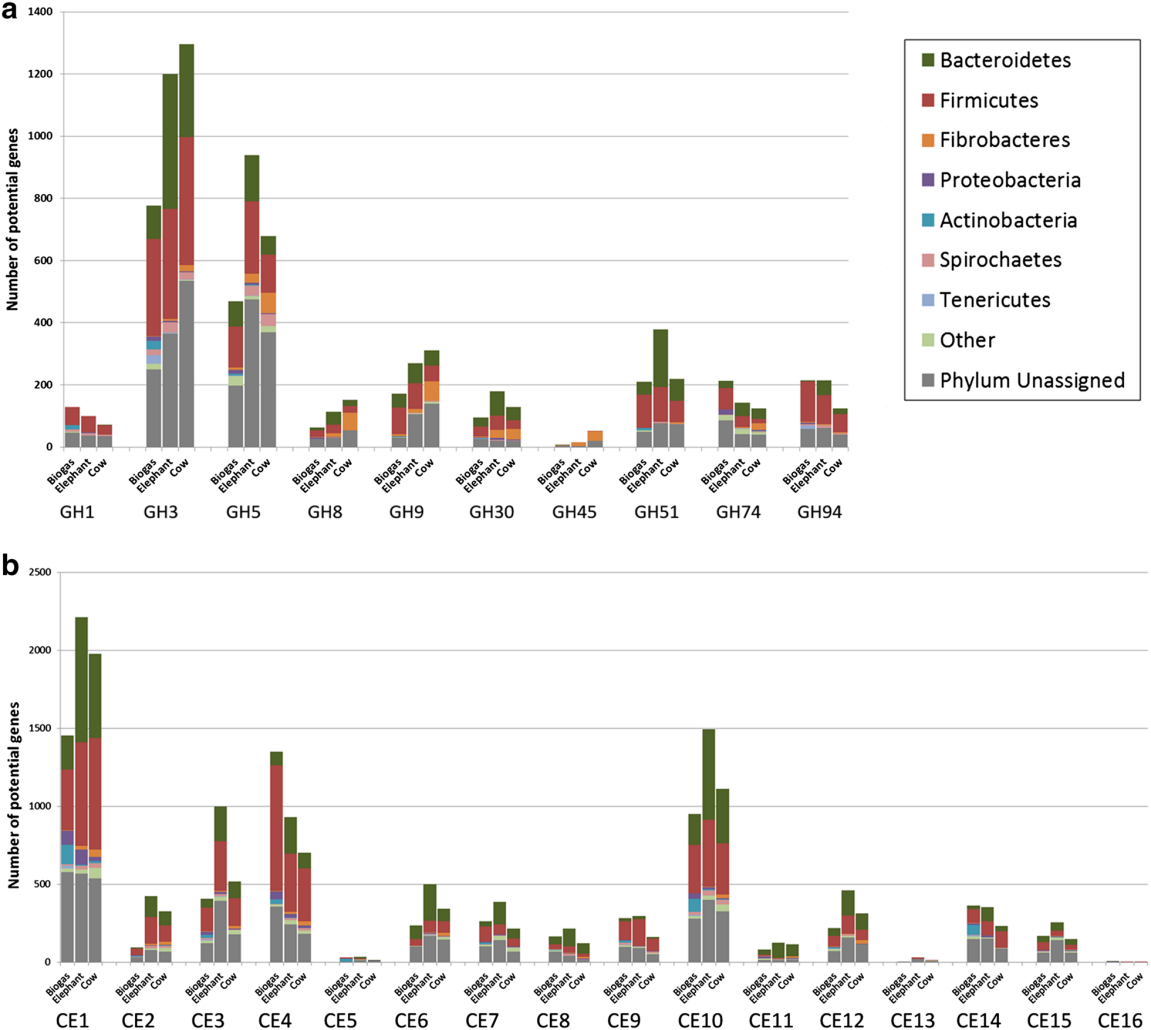
Abundance and taxonomic origin of hydrolytic enzymes in the biogas, elephant, and cow rumen data sets. **a** Dominant cellulolytic glycoside hydrolase families and **b** carbohydrate esterases families in metagenomic data sets. Sequences which could not be assigned to a distinct bacterial phylum using MEGAN’s LCA algorithm are shown in *grey*. The number of potential enzymes was normalized to 1 Gb of assembled DNA

In a next step, we assigned the taxonomic origin to the identified potential cellulolytic GH encoding genes via a protein blast search against the NCBI non-redundant database in combination with the MEGANs LCA algorithm. Using this approach, we were able to elucidate the phyla which contributed most to the hydrolytic metagenomic potential in the fermenter. We found that most of the predicted enzymes belonging to the cellulolytic GH families 1, 3, 5, 8, 9, 30, 45, 51, 74 and 94 showed the highest coverage for affiliates of the *Firmicutes* in the biogas fermenter (Fig. 2a). Notably, the number of predicted enzymes affiliated with the phylum of the *Bacteroidetes* was much lower in the analyzed cellulolytic GH families. A small fraction of predicted enzymes originated from the phyla *Actinobacteria,Spirochaetes*, and *Tenericutes*.

A similar analysis was done for the CE family genes and enzymes. CE family enzymes are mainly carbohydrate-active esterases and were recently introduced into the CAZy database. Within our data set, predicted genes for all 16 known CE families were covered and a total of 7655 genes possibly encoding for CE family enzymes were identified. This equals 6100 CEs per Gb of assembled DNA. The most predominant families were 1, 4, and 10. Thereby, we observed 1826 hits for family CE1, 1697 hits for family CE4, and 1198 hits for family CE10. Altogether these findings implied a high GH and CE enzyme diversity within the data set and suggested that the majority of the genes coding for these enzymes were derived from the *Firmicutes*.

To relate the above-made findings to other highly cellulolytic microbial communities, we compared these values to published and very comprehensive studies of two natural systems. The first data set was derived from a microbiome adherent to switchgrass, which was incubated in the rumen of a cow for 72 h [23]. The second data set was obtained from the feces of an adult Asian zoo elephant and published in 2014 by our group [15]. Both of these assembled metagenomic data sets were of a similar size as the data set generated in this study from an agricultural biogas plant. These data sets are, to our knowledge, the largest data sets of cellulolytic communities publicly available so far. In the original studies, both samples were described as being highly diverse and rich in hydrolytic enzymes. Within this framework, it is noteworthy that cows and elephants rely on different strategies for the digestion of their food. Elephants are hindgut fermenters and degrade their diet in the caecum, whereas cows are foregut fermenters and digest their food in the rumen [61]. While cows mainly fed on grass, elephants digest a wider spectrum of plant-derived biomass, and as a consequence, their microbiomes are, in part, different.

**Fig. 3:**
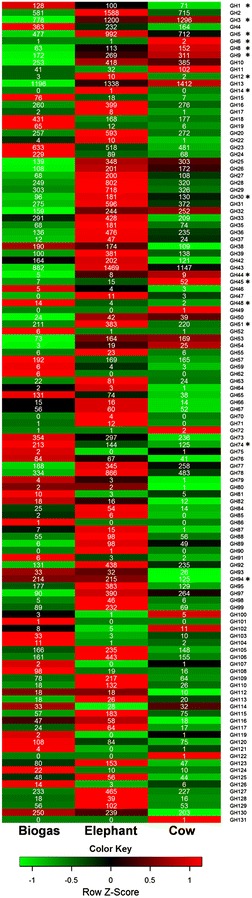
Heatmap indicating the abundance and distribution of potential GH family enzymes in the assembled metagenomic data sets. Rows are color coded according to *Z*-score. A *Z*-score change of +1 is equal to one standard deviation above the row mean. GH families involved in cellulose degradation are labeled with an *asterisk*. GH families not listed were not observed in the data sets. The amount of potential enzymes was normalized to 1 Gb of assembled DNA

The two published data sets were downloaded from IMG/MER (https://www.img.jgi.doe.gov/er/) and used for the comparative analyses with respect to the diversity and abundance of GH and CE enzymes (Table 4). We identified 22.5 GHs/Mbp of assembled DNA in the Elephant feces data set and 14.9 in the Cow data set compared to 13.8 in the biogas data set. For this comparison, the quantity of potential GH/CE encoding genes was analyzed independently of contig coverage. The abundance and distribution of the predicted genes coding for GH family enzymes in 1 Gb of assembled DNA are shown in Fig. 3. In a next step, the genes possibly coding for GH family enzymes involved in the breakdown of cellulose were examined in detail. For this, we included the 9 most abundant cellulolytic GH families which were observed in the biogas data set and compared their abundance and taxonomic origin in all three data sets (Fig. 2a). Interestingly, out of the 9 analyzed cellulolytic GH families, the biogas data set revealed the lowest number of potential enzymes in 7 GH families. Our analysis suggests that the lower abundance of GHs in these families can be attributed to a partial lack of enzymes derived from bacteria affiliated with *Fibrobacteres* and, especially, *Bacteroidetes*. Since both of these phyla comprise important polysaccharide-degrading bacteria in the gut and rumen of animals [12], an underrepresentation of genes coding for GHs derived from these groups implies a potentially important limitation in the biogas fermenter with regard to the hydrolysis of biomass.

**Table 4:** General overview on the metagenomic and hydrolytic potential of the analyzed biogas microbial community in relation to two other studies

Metagenome studied	Total DNA in assembly (Gbp)	DNA assembled^a^ (Gbp)	No. of predicted ORFs	No. of predicted GHs	No. of predicted CEs	GHs/Mbp	CEs/Mbp	Cellulolytic GHs/Mbp^c^	Ratio cellulolyti GHs *Firmicutes*/*Bacteroidetes* ^c^	Ratio CEs *Firmicutes*/*Bacteroidetes*	Data source
Agricultural biogas plant fermenter	58.7	1.25	2,011,807	17,305	7655	13.8	6.1	2.5	2.8:1	2.4:1	IMG ID: 3300002898 This study
Elephant feces from zoo animal	54.7	0.92	1,005,402	20,705	8344	22.5	9.1	3.8	1: 1	0.9:1	IMG ID: 3300001598 [15]
Cow rumen, switch grass	111.4	1.55^b^	2,083,556	23,110	9891	14.9	6.4	3.2	1.4:1	1.3:1	IMG ID: 2061766007 [23]

^a ^ DNA in this study was assembled with the IDBA-UD assembler; DNA in the studies by Ilmberger [15] and Hess [23] was assembled using velvet

^b^ Uncalled nucleotides (Ns) from scaffolds not included

^c^ GH families included: GH1, GH3, GH5, GH6, GH8, GH9, GH12, GH14, GH30, GH44, GH45, GH48, GH51, GH74 and GH94

With respect to the carbohydrate esterase family enzymes, a similar observation was made. Of the 16 families analyzed, only the CE families 4 and 14 had equal or more hits in the biogas data set compared to the elephant feces and cow rumen data sets (Fig. 2b). Distinct differences in the abundance of potential CEs were observed in the families CE1, CE2, CE3 and CE10. Enzymes assigned to these families share diverse enzymatic activities and substrate specificities, including acetyl xylan esterases and feruloyl esterases. Both of these groups of enzymes have been shown to be important accessory enzymes involved in the degradation of lignocellulosic biomass [62, 63]. A decreased overall diversity of CEs in the biogas fermenter might point to a disadvantage in the ability to efficiently degrade biomass compared to the two natural systems.

In addition to the identification of potential GHs and CEs encoding genes, we wanted to assess the presence of cellulosome encoding gene clusters in the different metagenome data sets. For this analysis, we used amino acid sequences of known cellulosomal scaffolding proteins and an iterative protein sequence similarity search with a cut-off score of 700. We identified a total of 3 hits in the biogas fermenter metagenome data set, 2 hits in the cow rumen data set, and no hits in the elephant feces data set (Additional file 5: Table S6). This analysis demonstrates a reduced diversity of cellulosome-producing bacteria in the cow rumen data set and, possibly, a complete lack of cellulosomes in the elephant feces data set. The potential cellulosomal scaffolding encoding genes, which were identified in the biogas data set, were allocated to the bacterial genome bins 96, pb35-1, and pb235-1. While the bin 96 was assigned to the genus C*lostridium,* the bins pb35-1 and pb235-1 were both assigned to the family *Lachnospiraceae.* Interestingly, a nucleotide blast search of the putative cellulosomal scaffolding genes found in the bin pb35-1 showed a 99 % identity to the recently described thermophilic cellulose-degrading bacterium *Herbinix hemicellulosilytica* which was isolated from a thermophilic biogas reactor [64, 65]. This finding suggests that this organism is also present in the community of our sampled fermenter and indicates that this species produces cellulosomes.

### RNA-Seq identifies metabolically highly active bacterial and archaeal groups as well as highly transcribed genes in the biogas fermenter

In the light of the above-made findings, we asked which families of GH enzymes were highly transcribed in the biogas fermenter at the time of sampling. In addition, we wanted to know, whether the highly transcribed genes were affiliated mainly with the *Bacteroidetes*, the *Firmicutes,* or other phyla, and relate our findings to a natural cellulolytic system. For this, we conducted RNA-Seq of a biogas fermenter sample taken from the same biogas plant at a later time point and an elephant feces sample. Because the initial elephant feces data set was published by our group, we had access to samples from the same animal, as described in the original publication by Ilmberger et al. [15]. Due to difficulties obtaining a comparable sample as analyzed by Hess and colleagues [23], we did not include the cow rumen in the RNA-Seq-based analysis. RNA extraction, sample preparation, sequencing, and data processing are described in “Methods” section. An overview about the processing steps and the number of cDNA sequence reads obtained for both samples is provided in Table 5.

**Table 5:** RNA-Seq processing steps and number of cDNA sequence reads obtained for elephant feces and biogas fermenter samples

Processing step		Elephant	Biogas
Pre-processing	No. of input reads	141,700,987	282,930,624
	After removal of polyA tails and short sequences <99	103,591,215	282,711,062
SortMeRNA	After rRNA removal	77,904,289	274,661,662
Bowtie2	Input reads	77,904,289	274,661,662
	Reads aligned 0 times	62,293,074 (79.96 %)	49,200,963 (17.91 %)
	Reads aligned exactly 1 time	12,328,533 (15.85 %)	172,293,650 (62.73 %)
	Reads aligned >1 times	3,282,682 (4.21 %)	53,167,049 (19.36 %)
	Overall alignment rate	20.04 %	82.09 %
HTSeq-count	Counted	8,140,194 (52.14 %)	159,743,444 (70.85 %)
	No feature	3,784,252 (24.24 %)	37,124,802 (16.47 %)
	Ambiguous	382,196 (2.45 %)	6,133,988 (2.72 %)
	Too low alignment quality (MAPQ <10)	3,304,573 (21.17 %)	22,458,465 (9.96 %)

In a first step, we wanted to get a general idea of the functional and taxonomic affiliation of highly transcribed genes in the sampled biogas fermenter. To do so, we examined the 100 ORFs with the highest levels of absolute transcripts via a protein blast search against the non-redundant NCBI protein database. It is important to state that this does not necessarily mean that these ORFs, seen individually, are the highest expressed ones. For 94 ORFs, homologs in the non-redundant protein data base were observed, while for the remaining 6 predicted ORFs no homologies were observed at all (Additional file 5: Table S5). The largest fraction of 31 ORFs showed the highest similarity to hypothetical proteins. Thus, this result might suggest that many gene functions of the microbial community in biogas fermenters are not well characterized. Of the ORFs with an assigned function, 20 ORFs were affiliated with bacterial ABC transporter substrate-binding proteins, and 13 ORFs scored the best hit for archaeal enzymes involved in methanogenesis. A large fraction of these methanogenesis-related ORFs encoded for different subunits of the Methyl-coenzyme M reductase.

Furthermore, 85 ORFs could be taxonomically classified using MEGANs LCA method. Of these, 67 ORFs were of bacterial origin and 17 of archaeal. The bacterial ORFs mainly originated from the *Firmicutes* (50) and within this phylum from the genus *Clostridia* (38). Notably, a large fraction of 23 ORFs was assigned to uncultured bacteria of the family *Peptococcaceae* suggesting a very high metabolic activity of these physiologically diverse and partly acetogenic bacteria [66] in our sampled fermenter. In addition, 6 ORFs were assigned to the order *Halanaerobiales*. The majority of species in the order *Halanaerobiales* is known for sugar fermentation or homoacetogenesis [67]. These results might indicate a high relevance of these two bacterial groups for acidogenesis and acetogenesis in this biogas fermenter and perhaps for agricultural biogas reactors in general. Most of the archaeal ORFs were assigned to the class *Methanomicrobia* (9) and within this class to the genus *Methanoculleus* (5). Two highly transcribed ORFs originated from the hydrogenotrophic methanogen *Methanoculleus bourgensis.* This finding is in accordance with recent research suggesting a predominant methanogenesis via the hydrogenotrophic pathway in agricultural biogas plants [68, 69]. Finally, two highly transcribed ORFs were attributed to yet uncultured archaea.

### RNA-Seq data imply that highly transcribed GH encoding genes in the biogas fermenter mainly originate from the Firmicutes, while bacteroidetal GH encoding genes are most transcribed in the elephant feces

**Fig. 4:**
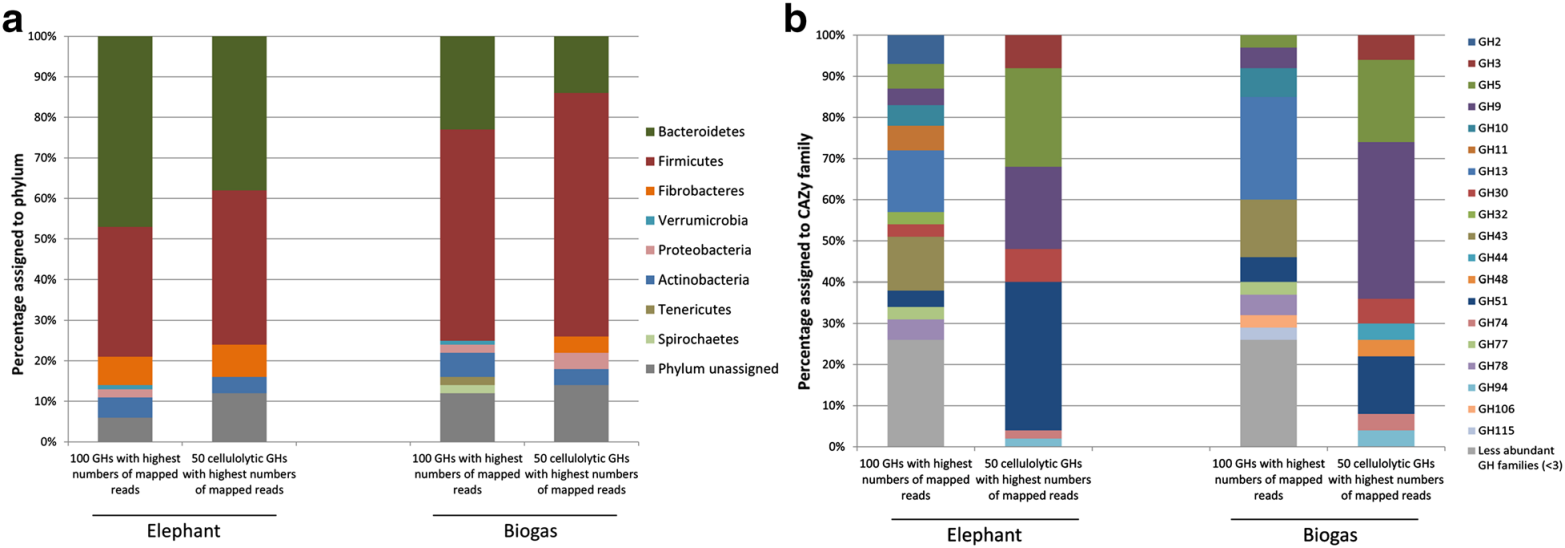
**a** Taxonomic origin and **b** CAZy family distribution of 100 GHs and 50 cellulolytic GHs with highest numbers of mapped cDNA reads obtained from RNA-Seq of a biogas fermenter sample in relation to an elephant feces sample. Cellulolytic GHs include the families: GH1, GH3, GH5, GH6, GH8, GH9, GH12, GH14, GH30, GH44, GH45, GH48, GH51, GH74 and GH94. For this analysis, Megan LCA parameters were adjusted to “top percent 40” and “LCA percent 50” for assignment of phyla

In a next step, we wanted to examine differences in the transcription of GHs enzymes in the biogas and elephant samples in detail. For this purpose, we restricted our RNA-Seq data analysis to ORFs, which were previously identified to potentially encode for GHs. Thereby, we examined the taxonomic origin and CAZy family distribution of 100 GHs (including all CAZy families) and 50 GHs (including only cellulolytic CAZy families) with the highest numbers of mapped cDNA reads. We found that the majority of GHs in both of these groups was affiliated with the *Firmicutes* in the biogas fermenter sample (Fig. 4a). The ratios of GHs derived from the *Firmicutes* versus the *Bacteroidetes* were 2.3:1, including all families and 4.3:1, including only the cellulolytic families. These results further supported the observation that the *Firmicutes* are the predominant group responsible for the hydrolysis of biomass in the biogas fermenter. In contrast, the same analysis conducted for the elephant feces indicated that most of the highly transcribed GHs originated from the *Bacteroidetes*. Here, the ratios of GHs derived from the *Firmicutes* versus the *Bacteroidetes* were 0.68:1, including all families and 1:1, including only the cellulolytic families. Compared to the biogas fermenter, a larger fraction of GHs was also affiliated with the *Fibrobacteres*.

Furthermore, when examining the CAZy family distribution of the 50 cellulolytic GHs with the highest levels of absolute transcript, we observed that in the elephant feces, most of the putative enzymes were assigned to the family GH51 followed by GH5 and GH9 (Fig. 4b). In the biogas fermenter, the GH9 family was most frequently observed followed by GH5 and GH51. While these three GH families are all involved in the hydrolysis of lignocellulose, they differ in their substrate specificities. Interestingly, the GH 51 family contains many hemicellulases while the GH9 family mainly includes cellulose-specific enzymes. Altogether these results supported the notion that there are distinct differences in the cellulolytic bacteria and enzymes involved in the degradation of lignocellulosic biomass between the biogas fermenter and the elephant feces sample. In the biogas fermenter, highly transcribed cellulolytic GHs were four times more often affiliated with the *Firmicutes* compared to the *Bacteroidetes* (ratio 4.3:1), while an almost equal distribution of these enzymes was observed in the elephant feces sample (ratio 1:1).

### RNA-Seq identifies transcription of cellulolytic GH family enzymes in the bacterial bins generated from the biogas fermenter metagenome

**Fig. 5:**
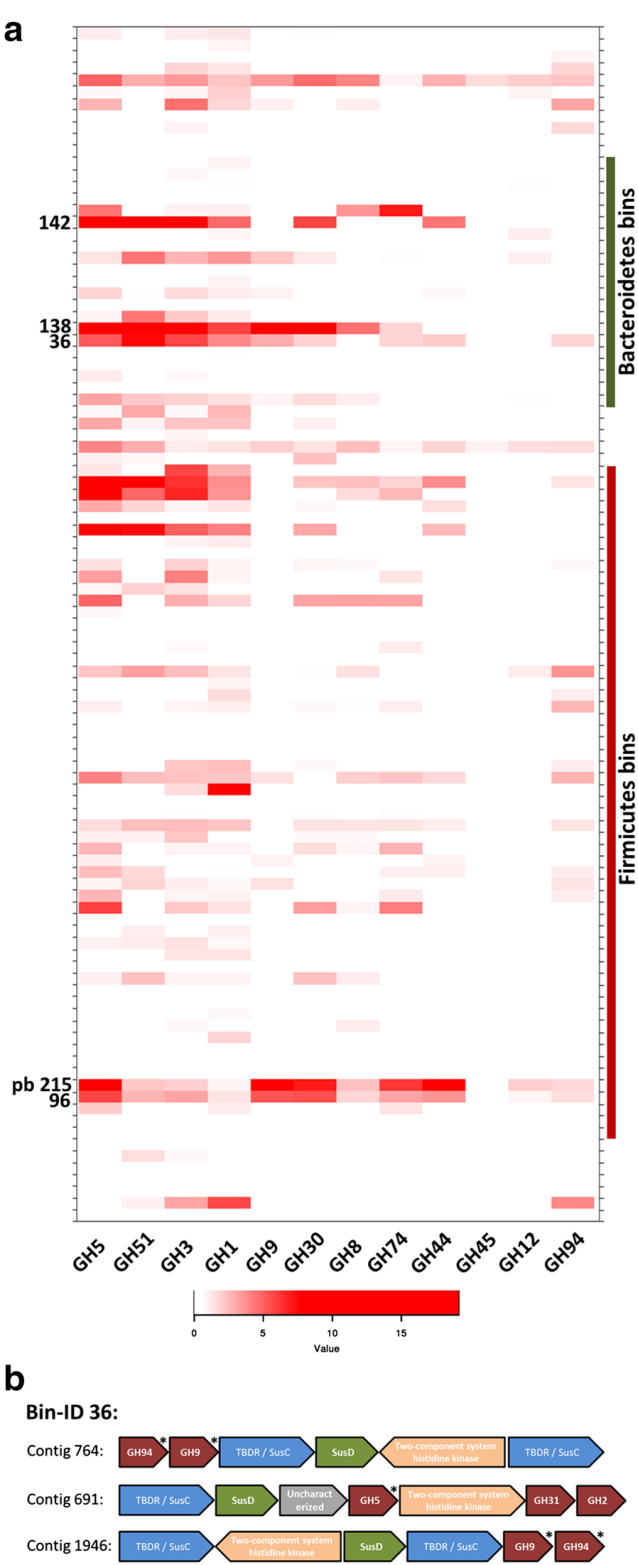
**a** Heatmap reflecting the expression of cellulolytic GH families in the bacterial bins generated from the biogas fermenter metagenome. Expression strength is shown as square root of the rpkm value. Bin-IDs for selected bins affiliated with the *Bacteroidetes* and the *Firmicutes* are indicated. All other bin-IDs are given in Additional file 9 with a continuous labeling. **b** Physical map of three putative cellulolytic PULs. The PULs were identified on three contigs assigned to the bacteroidetal bin 36. TBDR = TonB-dependent receptor. An *asterisk* indicates GH families with cellulase (GH5, GH9) or cellobiase (GH94) activity

To further investigate the transcription of cellulolytic GH families in individual organisms in the biogas fermenter, we utilized the binned bacterial contigs which were generated from the biogas fermenter metagenome. For this, RNA-Seq data were mapped onto the binned bacterial contigs (Fig. 5a). Although the binned DNA represented only a part of the complete metagenome, our analysis shows that in individual bacteroidetal genome bins, multiple cellulase-encoding genes were strongly transcribed (e.g. Bin-IDs 36, 138 and 142). An in-depth analysis of these three bins resulted in the identification of multiple PULs, including three putatively cellulolytic PULs (Fig. 5b). In these PULs, cellulase-encoding genes were identified next to or in close proximity to an *SusC* and *SusD* gene pair. Interestingly, a two-component system histidine kinase gene was also found in all putatively cellulolytic PULs. The presence of this regulatory system might suggest a differential expression of PUL associated genes and enzymes in response to the respective “target” polymer. An induction of PUL gene transcription in response to specific plant polysaccharides was already shown for *B. ovatus* and *B. thetaiotaomicron* [70]. The identification of high transcription levels of cellulase-encoding genes in the bacteroidetal genome bin 36, together with the existence of potential cellulolytic PULs in the same bin, might provide an explanation how the hydrolysis of biomass is carried out by cellulolytic bacteroidetal species. In our opinion, this finding further supports the notion that the importance and potential of bacteroidetal organisms for the degradation of biomass in biogas fermenters were most likely under-estimated in the past.

This analysis also demonstrates and confirms the predominant expression of cellulases by various *Firmicutes*. Not surprisingly, numerous organisms belonging to the class Clostridia showed transcription of cellulolytic enzymes. Particularly, high levels of transcription of cellulolytic enzymes were observed for the clostridial bins 96 and pb215. The genome bin 96 was taxonomically classified as *Clostridium thermocellum* and in agreement with this classification, the cellulosomal-scaffolding protein A of *C. thermocellum* was identified in this bin (Additional file 5 : Table S6). The bin pb215 was classified as a not further specified *Ruminiclostridium*.

## Conclusion

In this paper, we provide evidence that the analyzed biogas fermenter contains a relatively lower abundance of glycoside hydrolases and carbohydrate esterases involved in the breakdown of lignocellulosic biomass as compared to two natural plant biomass degrading systems. This difference can be attributed to a partial lack of enzymes derived from bacteria affiliated with the *Fibrobacteres* and, especially, the *Bacteroidetes*. The partial deficiency of these enzymes implies a potentially important limitation in the biogas fermenter with regard to the initial hydrolysis of biomass. In addition, we were able to show that the mean ratio of the phyla *Firmicutes* vs *Bacteroidetes* is close to 1:1 in various fecal or gut microbiomes of herbivorous animals, while the *Bacteroidetes* were usually 5–6 times less prominent in the mainly agricultural biogas fermenters listed in Table 2.

In accordance with this observation, RNA-Seq data showed that highly transcribed cellulolytic GHs in the biogas fermenter were four times more often affiliated with the *Firmicutes* compared to the *Bacteroidetes,* while an equal distribution of these enzymes was observed in an elephant feces sample. Finally, we hypothesize that by finding ways to alter the ratio of the *Firmicutes* vs. the *Bacteroidetes* in favor of the *Bacteroidetes*, an increase in the overall hydrolytic performance of biogas plants might be achieved. This can potentially be realized by adding bacteriodetal isolates at high levels. However, it is likely that the added bacteroidetal organisms are quickly outcompeted again due to their better adaptation to natural habitats. To achieve a lasting increase in the abundance of the *Bacteroidetes* in biogas fermenters, the process conditions would have to be altered in a way that favors growth of this bacterial phylum. Consequently, further research is required to identify these conditions and factors, particularly as the microbiomes in natural systems are actively influenced and shaped by the host.

## Additional files

10.1186/s13068-016-0534-x Compressed rar file containing the bins generated from the biogas fermenter metagenome, part 1 of 4.
10.1186/s13068-016-0534-x Compressed rar file containing the bins generated from the biogas fermenter metagenome, part 2 of 4.
10.1186/s13068-016-0534-x Compressed rar file containing the bins generated from the biogas fermenter metagenome, part 3 of 4.
10.1186/s13068-016-0534-x Compressed rar file containing the bins generated from the biogas fermenter metagenome, part 4 of 4.
10.1186/s13068-016-0534-x Process parameters of the biogas fermenter at time of sampling. These data were kindly determined and provided by Bonalytic GmbH. **Table S2.** Diversity and richness of biogas fermenter samples taken in March and May 2013 based on 16S rRNA gene analysis. OTUs were clustered at 99% sequence similarity level and singleton OTUs (i.e. OTUs with only one sequence assigned to them) were removed. **Table S3.** Shared OTUs between both biogas fermenter samples. **Table S4.** Bin overview (tax. Assignment, completeness, contamination, other assembly stats). **Table S5.** Blastp results and MEGAN LCA assignement of 100 ORFs with the highest numbers of mapped reads in the biogas fermenter. **Table S6.** Results of an iterative protein sequence search using Jackhmmer (hmmer 3.1 package) to identify potential cellulosomal scaffoldin proteins in the metagenomic datasets using a score cutoff of 700.
10.1186/s13068-016-0534-x Additional TEM pictures of biogas fermenter content.
10.1186/s13068-016-0534-x Comparison of total cellulolytic activities in biogas fermenter content and feces samples of various herbivorous animals determined via 3,5-dinitrosalicylic acid (DNS) assay.
10.1186/s13068-016-0534-x Compressed rar file containing four krona plots. The krona plots illustrate the results of the 16S rRNA gene amplicon analysis conducted for the March and May biogas fermenter samples.
10.1186/s13068-016-0534-x High-resolution version of Fig. 5a with continuous labeling of all bacterial bins. Bin-IDs are color coded according to assigned phylum.

## Abbreviations

CAZy: carbohydrate-active enzyme
CE: carbohydrate esterase
CMC: carboxymethycellulose
CTAB: cetyltrimethylammoniumbromid
DMSO: dimethylsulfoxid
DNS: 3,5-dinitrosalicylic acid
GH: glycoside hydrolase
HMM: hidden Markov model
LCA: lowest common ancestor
ORF: open reading frame
OTU: operational taxonomic unit
PULs: polysaccharide utilization loci
TEM: transmission electron microscopy
