# Supplementary material for: Deep metagenome and metatranscriptome analyses of microbial communities affiliated with an industrial biogas fermenter, a cow rumen, and elephant feces reveal major differences in carbohydrate hydrolysis strategies
Source: Biotechnol Biofuels. 2016 Jun 7;9:121. doi: 10.1186/s13068-016-0534-x (PMC4897800; doi:10.1186/s13068-016-0534-x)
Supplement: Supplementary file 5 — 10.1186/s13068-016-0534-x Process parameters of the biogas fermenter at time of sampling. These data were kindly determined and provided by Bonalytic GmbH. Table S2. Diversity and richness of biogas fermenter samples taken in March and May 2013 based on 16S rRNA gene analysis. OTUs were clustered at 99% sequence similarity level and singleton OTUs (i.e. OTUs with only one sequence assigned to them) were removed. Table S3. Shared OTUs between both biogas fermenter samples. Table S4. Bin overview (tax. Assignment, completeness, contamination, other assembly stats). Table S5. Blastp results and MEGAN LCA assignement of 100 ORFs with the highest numbers of mapped reads in the biogas fermenter. Table S6. Results of an iterative protein sequence search using Jackhmmer (hmmer 3.1 package) to identify potential cellulosomal scaffoldin proteins in the metagenomic datasets using a score cutoff of 700. [file 13068_2016_534_MOESM5_ESM.pdf]

Table S1: Process parameters of the biogas fermenter at time of sampling. These data were kindly determined and provided by Bonalytic GmbH.

Sampling date	pH	EC [mS/cm]	VOA [g HAceq/l]	TIC [g CaCO3/l]	VOA/TIC ratio	NH4+-N [g/l]	DM [g/kg]	ODM [g/kg]	Acetic acid [g/l]	Propionic acid [g/l]	Butyric acid [g/l]	Isobutyric acid [g/l]	Valeric acid [g/l]	Isovaleric acid [g/l]	Hexanoic acid [g/l]	Ca [g/kg dm]	K [g/kg dm]	Mg [g/kg dm]	Na [g/kg dm]	P [g/kg dm]	S [g/kg dm]
5-Mar-2013	7.9	35.6	3.28	18.7	0.18	3.14	103	78.3	0.13	< 0.03	< 0.03	< 0.03	< 0.03	< 0.03	< 0.03	20.7	47.6	7.03	4.61	10.4	5.97
27-May-2013	8.0	34.2	3.06	17.7	0.17	2.98	87.4	65.6	0.29	< 0.03	< 0.03	< 0.03	< 0.03	< 0.03	< 0.03	16.4	41.8	5.72	2.92	9.38	4.98

Sampling date	Al [mg/kg dm]	As [mg/kg dm]	B [mg/kg dm]	Ba [mg/kg dm]	Cd [mg/kg dm]	Co [mg/kg dm]	Cr [mg/kg dm]	Cu [mg/kg dm]	Fe [mg/kg dm]	Hg [mg/kg dm]	Li [mg/kg dm]	Mn [mg/kg dm]	Mo [mg/kg dm]	Nb [mg/kg dm]	Ni [mg/kg dm]	Pb [mg/kg dm]	Se [mg/kg dm]	Si [mg/kg dm]	Sn [mg/kg dm]	Sr [mg/kg dm]	V [mg/kg dm]	Zn [mg/kg dm]
5-Mar-2013	870	<15	29.1	26	0.23	2.16	3.91	66.3	4340	<4.2	3.18	313	5.82	<0.3	6.67	2.5	<15	342	<1.4	41.6	2.26	233
27-May-2013	799	<15	30.9	27.2	0.24	1.57	3.12	53.3	3400	<4.2	2.31	333	3.3	<0.3	4.19	1.48	<15	421	<1.4	39.9	1.61	285

EC: Electric conductivity
VOA: Volatile organic acids
Haceq: Acetic acid equivalent
TIC: Total inorganic carbon
NH4+-N: Ammonium nitrogen
DM: Dry matter
ODM: organic Dry matter

Table S2: Diversity and richness of biogas fermenter samples taken in March and May 2013 based on 16S rRNA gene analysis. OTUs were clustered at 99% sequence similarity level and singleton OTUs (i.e. OTUs with only one sequence assigned to them) were removed.

Sample	A)Observed OTUs	B)Coverage (%)	Shannon index (H')	Chao1
Bacteria biogas fermenter March 2013	994	90	6.5	1101
Archaea biogas fermenter March 2013	59	77	1.9	77
Bacteria biogas fermenter May 2013	1108	92	6.4	1206
Archaea biogas fermenter May 2013	95	97	2.2	98

A) Singleton OTUs not included B) Calculated by comparing number of observed OTUs witch Chao1 index

Table S3: Shared OTUs between both biogas fermenter samples.

Shared OTUs	Archaea May	Archaea March	Bacteria March	Bacteria May
Archaea May	95	52	0	0
Archaea March	52	59	0	0
Bacteria March	0	0	994	691
Bacteria May	0	0	691	1108

Table S4: Bin overview (tax. Assignment, completeness, contamination, other assembly stats)

**good bins**							
Bin-ID	Taxonomy (Consensus Score > 0.8)	completeness	contamination	Mb	N50 for contigs	number of contigs	length of largest contig (Kb)
pb121	Bacteria|Fibrobacteres/Acidobacteria group|Fibrobacteres|Fibrobacteria|Fibrobacterales|Fibrobacteraceae|Fibrobacter|Fibrobacter succinogenes	98.84	4.40	2.645	19,300	217	64.705
pb122	Bacteria|Fibrobacteres/Acidobacteria group|Fibrobacteres|Fibrobacteria|Fibrobacterales|Fibrobacteraceae|Fibrobacter|Fibrobacter succinogenes	97.74	2.85	2.109	11,855	259	36.831
pb172	Bacteria|Bacteroidetes/Chlorobi group|Bacteroidetes	96.24	2.07	2.501	19,077	233	59.910
pb190	Bacteria|Firmicutes|Clostridia	98.28	6.74	2.230	16,089	234	67.993
pb192-1	Bacteria|Firmicutes|Clostridia	96.77	1.61	2.250	129,429	36	296.725
pb212	Bacteria	98.31	6.78	2.485	22,750	210	66.286
pb215	Bacteria|Firmicutes|Clostridia|Clostridiales|Ruminococcaceae|Ruminiclostridium	100.00	8.77	3.177	45,291	144	104.223
pb31	Bacteria|Spirochaetes|Spirochaetia|Spirochaetales|Spirochaetaceae|Treponema	99.30	7.23	2.150	16,062	213	65.974
pb35-2	Bacteria|Firmicutes|Clostridia|Clostridiales|Lachnospiraceae|Lachnoclostridium|Lachnoclostridium phytofermentans	98.30	8.58	2.710	28,325	143	69.025
pb3	Bacteria|Firmicutes|Clostridia	96.61	2.97	2.357	33,655	139	110.330
pb40	Bacteria|Firmicutes|Clostridia	95.11	0.13	1.469	12,735	160	38.031
pb6-1	Bacteria	96.00	1.33	1.750	105,819	37	187.059
pb65	Bacteria|Firmicutes|Clostridia|Clostridiales	98.60	7.69	2.314	66,410	77	132.303
pb69	Bacteria|Bacteroidetes/Chlorobi group|Bacteroidetes|Bacteroidia|Bacteroidales|Porphyromonadaceae	96.24	3.73	1.741	17,355	181	54.027
pb76-1	Bacteria|Firmicutes|Clostridia|Clostridiales|Ruminococcaceae	96.55	8.62	1.767	21,374	134	79.794
pb80	Bacteria|Firmicutes	95.10	2.97	2.648	77,072	95	124.511
pb85	Archaea|Euryarchaeota|Methanomicrobia|Methanosarcinales|Methanosarcinaceae|Methanosarcina|Methanosarcina barkeri	99.84	0.03	3.431	70,311	111	156.221
pb88	Bacteria|Bacteroidetes/Chlorobi group|Bacteroidetes|Bacteroidia|Bacteroidales	96.24	1.08	3.549	17,475	336	126.620
pb90	Bacteria|Spirochaetes|Spirochaetia|Spirochaetales|Spirochaetaceae|Treponema	99.30	9.91	2.444	22,018	215	65.456
pb97	Bacteria|Firmicutes|Clostridia|Clostridiales|Ruminococcaceae	97.09	1.43	2.143	36,708	91	144.351
**nearly complete genome drafts**							
Bin-ID	Taxonomy (Consensus Score > 0.8)	completeness	contamination	Mb	N50 for contigs	number of contigs	length of largest contig (Kb)
107	Bacteria|Firmicutes|Clostridia|Clostridiales	91.03	1.34	2.012	67,725	71	217.998
121	Bacteria|Firmicutes|Clostridia	93.22	0.00	2.043	51,306	93	106.941
125	Bacteria|Bacteroidetes/Chlorobi group|Bacteroidetes	96.24	1.34	2.340	20,117	159	49.839
145	Bacteria|Bacteroidetes/Chlorobi group|Bacteroidetes|Bacteroidia|Bacteroidales|Porphyromonadaceae|Paludibacter|Paludibacter propionicigenes	98.92	0.54	2.932	88,660	86	228.162
159	Bacteria|Firmicutes|Clostridia|Clostridiales	97.90	3.85	2.319	56,632	73	267.533
184	Bacteria|Chlamydiae/Verrucomicrobia group|Verrucomicrobia	93.92	3.58	4.152	13,165	446	53.631
18	Bacteria|Firmicutes|Clostridia|Clostridiales|Ruminococcaceae|Mageeibacillus|Mageeibacillus indolicus	94.44	1.77	2.121	15,834	248	52.710
45	Bacteria|Bacteroidetes/Chlorobi group|Bacteroidetes	95.59	0.81	1.986	17,036	164	57.356
90	Bacteria|Firmicutes|Clostridia|Clostridiales|Ruminococcaceae	95.45	0.00	2.004	166,983	25	505.654
96	Bacteria|Firmicutes|Clostridia|Clostridiales|Ruminococcaceae|Ruminiclostridium|Ruminiclostridium thermocellum	97.65	2.57	3.255	16,777	281	70.785
pb186-2	Bacteria|Firmicutes|Clostridia|Clostridiales|Lachnospiraceae|Lachnoclostridium|Lachnoclostridium phytofermentans	90.72	2.06	2.106	39,480	70	118.937
pb205	Bacteria|Firmicutes|Clostridia|Clostridiales	94.38	4.20	2.087	55,007	78	142.135
pb233-1	Bacteria|Firmicutes|Clostridia|Clostridiales|Ruminococcaceae	93.96	1.68	2.000	46,118	69	91.208
pb235-2	Bacteria|Firmicutes|Clostridia|Clostridiales	93.17	4.79	2.018	17,760	148	54.200
pb237	Bacteria|Firmicutes|Clostridia|Clostridiales	90.03	2.82	1.655	8,072	296	45.030
pb35-1	Bacteria|Firmicutes|Clostridia|Clostridiales|Lachnospiraceae|Lachnoclostridium	91.05	1.34	2.288	51,148	66	138.353
pb47	Bacteria|Chlamydiae/Verrucomicrobia group|Verrucomicrobia	90.03	3.41	2.785	22,427	224	64.038
pb60-2	Bacteria|Firmicutes	92.58	2.54	2.729	106,815	75	322.125
pb61-1	Bacteria|Firmicutes|Clostridia|Clostridiales|Ruminococcaceae	93.10	0.67	1.733	9,928	227	41.862
pb84	Bacteria|Firmicutes|Bacilli|Bacillales|Bacillaceae|Oceanobacillus|Oceanobacillus iheyensis	92.88	1.90	2.351	14,552	208	82.982
**nearly complete pangenome drafts**							
Bin-ID	Taxonomy (Consensus Score > 0.8)	completeness	contamination	Mb	N50 for contigs	number of contigs	length of largest contig (Kb)
114	Bacteria|Firmicutes|Clostridia	90.80	19.75	2.456	14,476	308	58.016
118	Bacteria|Bacteroidetes/Chlorobi group|Bacteroidetes	96.55	8.62	1.717	7,498	293	21.498
120	Bacteria|Bacteroidetes/Chlorobi group|Bacteroidetes|Bacteroidia|Bacteroidales	98.57	5.86	3.371	107,165	113	535.634
122	Bacteria|Firmicutes	98.28	15.52	2.756	98,798	72	225.431
128	Bacteria|Firmicutes|Clostridia|Clostridiales	98.28	38.09	2.304	47,314	91	78.962
131	Bacteria|Firmicutes|Clostridia|Clostridiales	98.39	27.90	2.771	42,529	119	137.108
135	Bacteria|Bacteroidetes/Chlorobi group|Bacteroidetes|Bacteroidia|Bacteroidales	92.24	28.62	3.713	8,219	565	32.270
137	Bacteria|Firmicutes|Clostridia|Clostridiales|Ruminococcaceae	96.64	17.24	2.021	32,847	109	91.596
138	Bacteria|Bacteroidetes/Chlorobi group|Bacteroidetes|Bacteroidia|Bacteroidales	100.00	50.71	3.221	14,313	376	45.118
142	Bacteria|Bacteroidetes/Chlorobi group|Bacteroidetes|Bacteroidia|Bacteroidales	98.28	36.39	3.603	19,789	334	79.100
149	Bacteria|Bacteroidetes/Chlorobi group|Bacteroidetes|Bacteroidia|Bacteroidales	100.00	91.44	5.381	11,252	648	61.964
162	Bacteria|Firmicutes|Clostridia	94.23	13.46	2.647	74,664	90	259.569
165	Bacteria|Firmicutes|Clostridia|Clostridiales|Peptococcaceae|Pelotomaculum|Pelotomaculum thermopropionicum	94.94	11.18	2.864	56,541	99	205.407
167	Bacteria|Firmicutes|Clostridia|Clostridiales	99.30	16.08	2.243	50,875	72	155.488
16	Bacteria|Firmicutes|Clostridia	99.19	42.97	3.191	8,807	580	73.579
172	Bacteria|Firmicutes|Clostridia|Clostridiales	93.71	11.89	2.446	58,832	87	147.770
179	Bacteria|Actinobacteria|Actinobacteria|Micrococcales	100.00	61.99	5.944	12,829	692	107.971
17	Bacteria|Bacteroidetes/Chlorobi group|Bacteroidetes|Bacteroidia|Bacteroidales	100.00	98.78	6.802	50,520	301	351.131
180	Bacteria	98.67	31.39	2.400	45,126	108	115.296
188	Bacteria|Bacteroidetes/Chlorobi group|Bacteroidetes|Bacteroidia|Bacteroidales	94.05	19.03	2.278	40,218	171	117.004
200	Bacteria|Actinobacteria|Actinobacteria|Corynebacteriales|Corynebacteriaceae|Corynebacterium	100.00	56.62	4.101	17,663	505	157.817
201	Bacteria|Bacteroidetes/Chlorobi group|Bacteroidetes|Bacteroidia|Bacteroidales|Porphyromonadaceae|Paludibacter|Paludibacter propionicigenes	93.10	23.28	4.195	17,714	390	101.199
204	Bacteria|Firmicutes|Clostridia|Clostridiales|Ruminococcaceae|Mageeibacillus|Mageeibacillus indolicus	96.34	13.65	2.387	33,234	160	109.185
23	Bacteria	96.70	23.08	3.171	74,083	228	242.613
36	Bacteria|Bacteroidetes/Chlorobi group|Bacteroidetes|Bacteroidia|Bacteroidales	100.00	25.86	3.075	48,908	106	149.345
41	Bacteria	98.56	12.00	1.999	64,174	61	165.126
43	Bacteria|Firmicutes|Clostridia|Clostridiales	99.07	38.58	3.220	95,854	89	256.456
48	Archaea|Euryarchaeota|Methanobacteria|Methanobacteriales|Methanobacteriaceae|Methanobacterium	99.07	19.63	2.241	47,585	99	166.659
5	Bacteria|Firmicutes|Clostridia|Clostridiales|Ruminococcaceae	95.19	11.21	2.187	58,951	80	179.040
82	Bacteria|Firmicutes|Clostridia|Clostridiales	95.80	11.19	3.264	29,444	199	106.969
pb15-1	Bacteria	98.31	5.93	2.451	9,604	394	56.127
pb166-2	Bacteria|Bacteroidetes/Chlorobi group|Bacteroidetes	95.48	7.14	2.355	22,487	183	73.823
pb199	Bacteria	99.94	7.69	5.025	39.650	217	133,292
pb38-1	Bacteria|Tenericutes|Mollicutes|Acholeplasmatales|Acholeplasmataceae	92.06	6.33	1.123	24,983	80	54.497
pb70	Bacteria|Firmicutes|Clostridia|Clostridiales|Clostridiaceae|Alkaliphilus|Alkaliphilus oremlandii	91.13	5.20	2.413	28,556	201	209.858
pb78-1	Bacteria|Firmicutes	94.07	8.47	2.097	60,316	53	162.275
pb93-2	Bacteria|Firmicutes|Clostridia|Clostridiales|Syntrophomonadaceae	97.92	5.17	2.625	69,056	80	171.110
**incomplete genome drafts**							
Bin-ID	Taxonomy (Consensus Score > 0.8)	completeness	contamination	Mb	N50 for contigs	number of contigs	length of largest contig (Kb)
109	Bacteria|Firmicutes|Erysipelotrichia|Erysipelotrichales|Erysipelotrichaceae|Erysipelothrix|Erysipelothrix rhusiopathiae	79.90	4.04	1.120	5,151	279	19.234
141	Bacteria|Proteobacteria|delta/epsilon subdivisions|Deltaproteobacteria	73.99	2.58	2.545	5,013	674	26.602
147	Bacteria|Firmicutes|Clostridia	90.25	6.79	2.117	42,492	105	144.721
175	Bacteria|Spirochaetes|Spirochaetia|Spirochaetales|Spirochaetaceae|Sphaerochaeta	64.12	2.84	1.015	2,321	476	9.900
178	Bacteria|Bacteroidetes/Chlorobi group|Bacteroidetes|Bacteroidia|Bacteroidales	80.46	1.40	1.499	8,230	255	32.211
1	Bacteria|Firmicutes|Clostridia|Clostridiales	73.48	2.62	1.283	4,246	351	16.921
20	Bacteria|Firmicutes|Clostridia|Clostridiales|Ruminococcaceae	64.22	2.35	1.547	2,173	761	9.306
2	Bacteria|Firmicutes|Clostridia|Clostridiales|Ruminococcaceae	63.64	4.80	2.007	1,864	1116	8.839
33	Archaea|Euryarchaeota	71.64	2.26	1.172	2,027	615	6.708
3	Bacteria|Firmicutes|Clostridia|Clostridiales	61.88	5.44	1.007	2,137	507	7.779
49	Bacteria|Firmicutes|Clostridia|Halanaerobiales|Halanaerobiaceae	72.10	5.26	1.233	1,959	669	6.919
72	Bacteria|Fibrobacteres/Acidobacteria group|Acidobacteria	68.80	5.41	2.193	2,569	960	13.184
75	Bacteria|Firmicutes|Clostridia	71.08	3.04	1.380	2,916	553	11.961
pb108-3	Bacteria|Firmicutes|Clostridia	88.35	5.23	2.207	37,524	96	85.991
pb162	Bacteria|Firmicutes|Clostridia|Clostridiales	88.36	7.07	1.811	3,318	669	16.219
pb173	Bacteria	87.77	4.39	0.826	12,005	102	32.609
pb177	Bacteria|Firmicutes|Clostridia	88.45	3.06	2.110	5,461	512	27.057
pb185	Bacteria|Firmicutes|Clostridia|Clostridiales|Ruminococcaceae|Mageeibacillus|Mageeibacillus indolicus	84.97	4.18	1.728	4,443	513	15.446
pb206-2	Bacteria|Bacteroidetes/Chlorobi group|Bacteroidetes|Bacteroidia|Bacteroidales	81.97	5.41	2.138	11,327	239	47.401
pb207	Bacteria|Bacteroidetes/Chlorobi group|Bacteroidetes|Bacteroidia|Bacteroidales	87.50	0.50	2.513	11,906	302	38.600
pb233-3	Bacteria|Firmicutes|Clostridia|Clostridiales|Ruminococcaceae|Mageeibacillus|Mageeibacillus indolicus	84.14	4.49	1.413	11,939	166	47.543
pb235-1	Bacteria|Firmicutes|Clostridia|Clostridiales|Lachnospiraceae|Lachnoclostridium	86.35	4.36	2.336	71,225	50	181.381
pb243	Bacteria|Spirochaetes|Spirochaetia|Spirochaetales|Spirochaetaceae|Sphaerochaeta|Sphaerochaeta globosa	89.14	3.51	2.397	12,472	319	81.956
pb246	Bacteria|Firmicutes|Clostridia|Clostridiales|Ruminococcaceae	85.81	5.09	1.580	3,920	490	32.604
pb2	Bacteria|Bacteroidetes/Chlorobi group|Bacteroidetes|Bacteroidia|Bacteroidales	82.22	5.20	2.148	3,435	757	23.329
pb30	Bacteria|Planctomycetes|Planctomycetia|Planctomycetales|Planctomycetaceae	87.12	0.99	3.035	7,797	516	29.695
pb6-2	Bacteria	88.55	1.82	1.390	8,194	254	31.274

Table S5: Blastp results and MEGAN LCA assignement of 100 ORFs with the highest numbers of mapped reads in the biogas fermenter

<table>
<tr><th>ORF</th><th>Numer of mapped RNA reads</th><th>Top hit description</th><th>Hit Length</th><th>Max Score</th><th>Total Score</th><th>Query coverage (in %)</th><th>E-Value</th><th>Max. Identity</th><th>MEGAN 5
LCA taxonomic assignment</th></tr>
<tr><td>contig-80_19653_7</td><td>1072827</td><td>methyl-coenzyme M reductase subunit B [uncultured archaeon]</td><td>434</td><td>748.043</td><td>748.043</td><td>100</td><td>0</td><td>99</td><td>environmental samples <Archaea></td></tr>
<tr><td>contig-80_33866_1</td><td>930872</td><td>hypothetical protein [Methanoculleus bourgensis] >gi|396938205|emb|CCJ35460.1| hypothetical protein</td><td>518</td><td>615.15</td><td>615.15</td><td>103</td><td>0</td><td>67</td><td>Methanoculleus</td></tr>
<tr><td>contig-80_13497_1</td><td>856474</td><td>hypothetical protein AA931_10905 [Peptococcaceae bacterium 1109]</td><td>818</td><td>603.979</td><td>603.979</td><td>99</td><td>0</td><td>69</td><td>Peptococcaceae</td></tr>
<tr><td>contig-80_21488_7</td><td>820923</td><td>PTS mannitol IIC component [Enterococcus pallens] >gi|486865160|gb|EOH97972.1| PTS system, mannitol</td><td>586</td><td>725.702</td><td>725.702</td><td>100</td><td>0</td><td>62</td><td>Lactobacillales</td></tr>
<tr><td>contig-80_168684_1</td><td>795758</td><td>No hit</td><td></td><td></td><td></td><td></td><td></td><td></td><td>No hits</td></tr>
<tr><td>contig-80_58347_5</td><td>718705</td><td>methyl-coenzyme M reductase subunit A [uncultured archaeon]</td><td>568</td><td>752.281</td><td>752.281</td><td>100</td><td>0</td><td>100</td><td>Archaea</td></tr>
<tr><td>contig-80_23290_4</td><td>684024</td><td>extracellular solute-binding protein family 5 [Firmicutes bacterium CAG:449]</td><td>860</td><td>413.305</td><td>413.305</td><td>82</td><td>9.79E-127</td><td>37</td><td>environmental samples <clostridial firmicutes></td></tr>
<tr><td>contig-80_2468_2</td><td>560253</td><td>ABC transporter substrate-binding protein [Limnochorda pilosa]</td><td>576</td><td>482.256</td><td>482.256</td><td>94</td><td>5.37E-160</td><td>45</td><td>Halanaerobium</td></tr>
<tr><td>contig-80_89734_2</td><td>448685</td><td>formate dehydrogenase subunit alpha [Methanoculleus bourgensis] >gi|396939866|emb|CCJ37121.1| for</td><td>689</td><td>1335.09</td><td>1335.09</td><td>99</td><td>0</td><td>90</td><td>Methanoculleus</td></tr>
<tr><td>contig-80_71905_1</td><td>448196</td><td>hypothetical protein AA931_11200 [Peptococcaceae bacterium 1109]</td><td>555</td><td>957.592</td><td>957.592</td><td>99</td><td>0</td><td>81</td><td>Peptococcaceae</td></tr>
<tr><td>contig-80_58347_4</td><td>439480</td><td>methyl-coenzyme M reductase subunit G [uncultured archaeon]</td><td>253</td><td>525.783</td><td>525.783</td><td>97</td><td>0</td><td>99</td><td>Archaea</td></tr>
<tr><td>contig-80_3126_21</td><td>426197</td><td>hypothetical protein AA931_00620 [Peptococcaceae bacterium 1109]</td><td>461</td><td>157.147</td><td>157.147</td><td>105</td><td>6.77E-39</td><td>29</td><td>Peptococcaceae</td></tr>
<tr><td>contig-80_3861_2</td><td>397581</td><td>hypothetical protein LIP_1185 [Limnochorda pilosa]</td><td>3410</td><td>2801.16</td><td>3385.0382</td><td>95</td><td>0</td><td>51</td><td>Not assigned</td></tr>
<tr><td>contig-80_33291_1</td><td>396617</td><td>hypothetical protein AA931_00290 [Peptococcaceae bacterium 1109]</td><td>818</td><td>139.043</td><td>139.043</td><td>109</td><td>1.26E-31</td><td>30</td><td>Peptococcaceae</td></tr>
<tr><td>contig-80_201192_1</td><td>378635</td><td>methylenetetrahydromethanopterin reductase [Methanoculleus bourgensis] >gi|396939342|emb|CCJ3659</td><td>330</td><td>623.624</td><td>623.624</td><td>99</td><td>0</td><td>95</td><td>Methanomicrobiaceae</td></tr>
<tr><td>contig-80_11213_1</td><td>370600</td><td>sugar ABC transporter substrate-binding protein [Limnochorda pilosa]</td><td>401</td><td>408.297</td><td>408.297</td><td>94</td><td>3.25E-136</td><td>54</td><td>Not assigned</td></tr>
<tr><td>contig-80_82829_2</td><td>355530</td><td>F420-dependent NADP reductase [Methanosphaerula palustris] >gi|219547574|gb|ACL18024.1| NADPH-d</td><td>226</td><td>316.62</td><td>316.62</td><td>99</td><td>1.21E-105</td><td>76</td><td>Methanosphaerula</td></tr>
<tr><td>contig-80_32942_2</td><td>353487</td><td>hypothetical protein AA931_11200 [Peptococcaceae bacterium 1109]</td><td>555</td><td>967.992</td><td>967.992</td><td>100</td><td>0</td><td>83</td><td>Peptococcaceae</td></tr>
<tr><td>contig-80_233686_1</td><td>346915</td><td>hypothetical protein AA931_10905 [Peptococcaceae bacterium 1109]</td><td>818</td><td>460.299</td><td>460.299</td><td>99</td><td>4.40E-152</td><td>64</td><td>Peptococcaceae</td></tr>
<tr><td>contig-80_201084_1</td><td>343033</td><td>hypothetical protein AA931_10905 [Peptococcaceae bacterium 1109]</td><td>818</td><td>408.297</td><td>408.297</td><td>99</td><td>2.28E-132</td><td>60</td><td>Peptococcaceae</td></tr>
<tr><td>contig-80_3956_1</td><td>337076</td><td>pyruvate-flavodoxin oxidoreductase [Clostridiales bacterium mt11]</td><td>1176</td><td>1831.61</td><td>1831.61</td><td>99</td><td>0</td><td>72</td><td>Clostridia</td></tr>
<tr><td>contig-80_183577_1</td><td>313105</td><td>hypothetical protein AA931_00290 [Peptococcaceae bacterium 1109]</td><td>818</td><td>164.851</td><td>164.851</td><td>106</td><td>6.48E-42</td><td>39</td><td>Peptococcaceae</td></tr>
<tr><td>contig-80_226378_1</td><td>306822</td><td>hypothetical protein [Erysipelotrichaceae bacterium NK3D112]</td><td>646</td><td>352.058</td><td>352.058</td><td>100</td><td>5.90E-112</td><td>52</td><td>Firmicutes</td></tr>
<tr><td>contig-80_8571_8</td><td>272301</td><td>hypothetical protein AA931_10905 [Peptococcaceae bacterium 1109]</td><td>818</td><td>1228.77</td><td>1228.77</td><td>99</td><td>0</td><td>71</td><td>Peptococcaceae</td></tr>
<tr><td>contig-80_38211_5</td><td>268888</td><td>hypothetical protein AA931_00290 [Peptococcaceae bacterium 1109]</td><td>818</td><td>161.77</td><td>161.77</td><td>100</td><td>8.97E-41</td><td>37</td><td>Peptococcaceae</td></tr>
<tr><td>contig-80_16430_1</td><td>259914</td><td>sugar ABC transporter substrate-binding protein [Peptococcaceae bacterium 1109]</td><td>329</td><td>572.392</td><td>572.392</td><td>99</td><td>0</td><td>87</td><td>Peptococcaceae</td></tr>
<tr><td>contig-80_48157_1</td><td>259644</td><td>hypothetical protein LIP_1185 [Limnochorda pilosa]</td><td>3410</td><td>1344.33</td><td>2128.1125</td><td>100</td><td>0</td><td>54</td><td>Not assigned</td></tr>
<tr><td>contig-80_4093_10</td><td>245182</td><td>ABC transporter substrate-binding protein [Limnochorda pilosa]</td><td>576</td><td>475.322</td><td>475.322</td><td>101</td><td>3.09E-157</td><td>43</td><td>Halanaerobium</td></tr>
<tr><td>contig-80_52000_1</td><td>227685</td><td>aldehyde oxidoreductase [Ruminococcus gauvreauii]</td><td>911</td><td>1488.01</td><td>1488.01</td><td>99</td><td>0</td><td>76</td><td>Clostridiales</td></tr>
<tr><td>contig-80_22025_2</td><td>218182</td><td>sugar ABC transporter substrate-binding protein [Limnochorda pilosa]</td><td>401</td><td>402.134</td><td>402.134</td><td>94</td><td>6.71E-134</td><td>53</td><td>Not assigned</td></tr>
<tr><td>contig-80_121403_1</td><td>208772</td><td>No hit</td><td></td><td></td><td></td><td></td><td></td><td></td><td>No hits</td></tr>
<tr><td>contig-80_58347_3</td><td>206726</td><td>methyl-coenzyme M reductase subunit C [uncultured archaeon]</td><td>207</td><td>410.223</td><td>410.223</td><td>99</td><td>4.06E-143</td><td>99</td><td>Archaea</td></tr>
<tr><td>contig-80_23408_1</td><td>203675</td><td>hypothetical protein AA931_12550 [Peptococcaceae bacterium 1109]</td><td>630</td><td>1219.91</td><td>1219.91</td><td>99</td><td>0</td><td>92</td><td>Peptococcaceae</td></tr>
<tr><td>contig-80_9517_9</td><td>194115</td><td>stage V sporulation protein S [Peptococcaceae bacterium 1109]</td><td>86</td><td>153.68</td><td>153.68</td><td>98</td><td>6.09E-46</td><td>89</td><td>Firmicutes</td></tr>
<tr><td>contig-80_25669_1</td><td>191007</td><td>ABC transporter solute-binding protein [Peptostreptococcaceae bacterium oral taxon 113] >gi|543981515|</td><td>445</td><td>523.472</td><td>523.472</td><td>92</td><td>7.65E-180</td><td>61</td><td>Bacteria</td></tr>
<tr><td>contig-80_61065_2</td><td>189587</td><td>hypothetical protein LIP_1185 [Limnochorda pilosa]</td><td>3410</td><td>288.5</td><td>1069.2004</td><td>88</td><td>1.10E-78</td><td>48</td><td>Not assigned</td></tr>
<tr><td>contig-80_21488_5</td><td>184607</td><td>mannitol-1-phosphate 5-dehydrogenase [Enterococcus raffinosus] >gi|486844990|gb|EOH78181.1| mannit</td><td>379</td><td>520.776</td><td>520.776</td><td>98</td><td>0</td><td>63</td><td>Lactobacillales</td></tr>
<tr><td>contig-80_312444_1</td><td>171786</td><td>Multiple sugar-binding periplasmic receptor ChvE [Herbinix sp. SD1D]</td><td>359</td><td>330.102</td><td>330.102</td><td>102</td><td>1.12E-108</td><td>63</td><td>Firmicutes</td></tr>
<tr><td>contig-80_55234_3</td><td>170774</td><td>Multiple sugar-binding periplasmic receptor ChvE [Herbinix sp. SD1D]</td><td>359</td><td>478.404</td><td>478.404</td><td>99</td><td>1.28E-165</td><td>70</td><td>Firmicutes</td></tr>
<tr><td>contig-80_11579_1</td><td>169839</td><td>hypothetical protein [Bacillus sp. JCE]</td><td>3124</td><td>487.649</td><td>3527.844</td><td>27</td><td>1.20E-134</td><td>34</td><td>Bacillales</td></tr>
<tr><td>contig-80_608665_1</td><td>168866</td><td>hypothetical protein AA931_00620 [Peptococcaceae bacterium 1109]</td><td>461</td><td>48.1358</td><td>48.1358</td><td>68</td><td>7.96E-04</td><td>36</td><td>Not assigned</td></tr>
<tr><td>contig-80_896734_1</td><td>168691</td><td>methyl CoM reductase alpha subunit, partial [uncultured methanogenic archaeon]</td><td>155</td><td>213.772</td><td>213.772</td><td>100</td><td>2.64E-68</td><td>100</td><td>Archaea</td></tr>
<tr><td>contig-80_52247_3</td><td>167803</td><td>ABC transporter substrate-binding protein [Anoxybacillus flavithermus]</td><td>558</td><td>397.897</td><td>397.897</td><td>97</td><td>2.08E-128</td><td>44</td><td>Bacteria</td></tr>
<tr><td>contig-80_58347_2</td><td>166943</td><td>methyl-coenzyme M reductase [Methanoculleus bourgensis] >gi|396939403|emb|CCJ36658.1| methyl-coe</td><td>158</td><td>278.485</td><td>278.485</td><td>99</td><td>1.14E-92</td><td>84</td><td>Methanoculleus</td></tr>
<tr><td>contig-80_52115_2</td><td>165024</td><td>membrane protein [Oribacterium parvum] >gi|394704654|gb|EJF12189.1| basic membrane protein [Oriba</td><td>401</td><td>443.736</td><td>443.736</td><td>93</td><td>1.33E-150</td><td>65</td><td>Firmicutes</td></tr>
<tr><td>contig-80_602_19</td><td>162919</td><td>sugar ABC transporter substrate-binding protein [Thermotoga maritima] >gi|15643361|ref|NP_228405.1|</td><td>419</td><td>252.292</td><td>252.292</td><td>102</td><td>3.44E-75</td><td>36</td><td>Bacteria</td></tr>
<tr><td>contig-80_90256_3</td><td>161415</td><td>hypothetical protein AA931_10905 [Peptococcaceae bacterium 1109]</td><td>818</td><td>244.202</td><td>244.202</td><td>98</td><td>1.35E-72</td><td>71</td><td>Peptococcaceae</td></tr>
<tr><td>contig-80_390436_1</td><td>156123</td><td>No hit</td><td></td><td></td><td></td><td></td><td></td><td></td><td>No hits</td></tr>
<tr><td>contig-80_61822_1</td><td>155928</td><td>ABC transporter substrate-binding protein [Peptococcaceae bacterium 1109]</td><td>408</td><td>661.759</td><td>661.759</td><td>95</td><td>0</td><td>78</td><td>Peptococcaceae</td></tr>
<tr><td>contig-80_39128_3</td><td>155666</td><td>aldehyde oxidoreductase [Ruminococcus gauvreauii]</td><td>911</td><td>1476.84</td><td>1476.84</td><td>99</td><td>0</td><td>76</td><td>Clostridiales</td></tr>
<tr><td>contig-80_5424_6</td><td>154426</td><td>Alcohol dehydrogenase, zinc-binding domain protein [Methanocorpusculum labreanum Z]</td><td>357</td><td>638.262</td><td>638.262</td><td>99</td><td>0</td><td>84</td><td>Methanocorpusculum</td></tr>
<tr><td>contig-80_1019865_1</td><td>152781</td><td>hypothetical protein AA931_10905 [Peptococcaceae bacterium 1109]</td><td>818</td><td>66.2402</td><td>66.2402</td><td>92</td><td>1.04E-10</td><td>64</td><td>Peptococcaceae</td></tr>
<tr><td>contig-80_6460_7</td><td>152727</td><td>sugar ABC transporter substrate-binding protein [Peptococcaceae bacterium 1109]</td><td>527</td><td>795.423</td><td>795.423</td><td>98</td><td>0</td><td>71</td><td>Clostridiales</td></tr>
<tr><td>contig-80_33866_4</td><td>150932</td><td>hypothetical protein BN140_0535 [Methanoculleus bourgensis MS2]</td><td>355</td><td>462.996</td><td>462.996</td><td>101</td><td>3.24E-159</td><td>64</td><td>Methanoculleus</td></tr>
<tr><td>contig-80_184701_1</td><td>149566</td><td>methyl-coenzyme M reductase [Methanoculleus bourgensis] >gi|396939402|emb|CCJ36657.1| methyl-coe</td><td>434</td><td>614.379</td><td>614.379</td><td>100</td><td>0</td><td>91</td><td>Archaea</td></tr>
<tr><td>contig-80_14342_7</td><td>144760</td><td>ABC transporter substrate-binding protein [Limnochorda pilosa]</td><td>576</td><td>479.174</td><td>479.174</td><td>101</td><td>9.41E-159</td><td>43</td><td>Halanaerobium</td></tr>
<tr><td>contig-80_6425_10</td><td>143590</td><td>sugar ABC transporter substrate-binding protein [Peptococcaceae bacterium 1109]</td><td>353</td><td>592.423</td><td>592.423</td><td>97</td><td>0</td><td>84</td><td>Clostridiales</td></tr>
<tr><td>contig-80_76725_1</td><td>140566</td><td>hypothetical surface-anchored protein [Coprobacillus sp. CAG:698]</td><td>1559</td><td>162.155</td><td>162.155</td><td>81</td><td>3.03E-37</td><td>28</td><td>Coprobacillus</td></tr>
<tr><td>contig-80_58347_1</td><td>139161</td><td>methyl-coenzyme M reductase subunit B [uncultured archaeon]</td><td>434</td><td>191.045</td><td>191.045</td><td>98</td><td>2.37E-56</td><td>100</td><td>environmental samples <Archaea></td></tr>
<tr><td>contig-80_517402_1</td><td>138608</td><td>hypothetical protein AA931_00620 [Peptococcaceae bacterium 1109]</td><td>461</td><td>58.151</td><td>58.151</td><td>102</td><td>3.05E-07</td><td>41</td><td>Peptococcaceae</td></tr>
<tr><td>contig-80_10299_6</td><td>135002</td><td>No hit</td><td></td><td></td><td></td><td></td><td></td><td></td><td>No hits</td></tr>
<tr><td>contig-80_72398_1</td><td>134486</td><td>peptidase S8 [Halanaerobium hydrogeniformans] >gi|311903494|gb|ADQ13935.1| peptidase S8 and S53 s</td><td>619</td><td>206.838</td><td>206.838</td><td>58</td><td>6.84E-55</td><td>40</td><td>Halanaerobiales</td></tr>
<tr><td>contig-80_7871_10</td><td>131473</td><td>pyruvate phosphate dikinase [Peptococcaceae bacterium 1109]</td><td>876</td><td>1468.37</td><td>1468.37</td><td>99</td><td>0</td><td>79</td><td>Peptococcaceae</td></tr>
<tr><td>contig-80_629047_1</td><td>129364</td><td>peptidase S8 [Halanaerobium hydrogeniformans] >gi|311903494|gb|ADQ13935.1| peptidase S8 and S53 s</td><td>619</td><td>97.0561</td><td>97.0561</td><td>90</td><td>2.48E-20</td><td>38</td><td>Halanaerobium</td></tr>
<tr><td>contig-80_179236_2</td><td>128304</td><td>transposase [[Clostridium] cellulolyticum] >gi|219999295|gb|ACL75896.1| transposase IS116/IS110/IS902</td><td>424</td><td>375.555</td><td>375.555</td><td>95</td><td>1.14E-124</td><td>62</td><td>Clostridiales</td></tr>
<tr><td>contig-80_213664_2</td><td>127979</td><td>No hit</td><td></td><td></td><td></td><td></td><td></td><td></td><td>No hits</td></tr>
<tr><td>contig-80_1058524_1</td><td>127279</td><td>hypothetical protein AA931_10905 [Peptococcaceae bacterium 1109]</td><td>818</td><td>102.449</td><td>102.449</td><td>100</td><td>5.31E-23</td><td>59</td><td>cellular organisms</td></tr>
<tr><td>contig-80_6458_4</td><td>126414</td><td>dipeptide-binding ABC transporter substrate-binding protein (TC 3.A.1.5.2) [Halanaerobium saccharolyticum</td><td>603</td><td>523.472</td><td>523.472</td><td>95</td><td>2.61E-175</td><td>45</td><td>Bacteria</td></tr>
<tr><td>contig-80_2203_3</td><td>125589</td><td>predicted solute binding protein [Bellilinea caldifistulae]</td><td>853</td><td>879.396</td><td>879.396</td><td>96</td><td>0</td><td>54</td><td>Bacteria</td></tr>
<tr><td>contig-80_507757_1</td><td>124932</td><td>peptidase S8 [Halanaerobium hydrogeniformans] >gi|311903494|gb|ADQ13935.1| peptidase S8 and S53 s</td><td>619</td><td>139.043</td><td>139.043</td><td>100</td><td>5.35E-35</td><td>46</td><td>Halanaerobiales</td></tr>
<tr><td>contig-80_29131_3</td><td>123233</td><td>lipoprotein [Defluviitoga tunisiensis] >gi|762220813|emb|CEP78642.1| lipoprotein [Defluviitoga tunisiensis</td><td>372</td><td>412.92</td><td>412.92</td><td>101</td><td>6.19E-139</td><td>55</td><td>Bacteria</td></tr>
<tr><td>contig-80_5759_12</td><td>121826</td><td>lipoprotein [Defluviitoga tunisiensis] >gi|762220813|emb|CEP78642.1| lipoprotein [Defluviitoga tunisiensis</td><td>372</td><td>414.846</td><td>414.846</td><td>98</td><td>8.18E-140</td><td>54</td><td>Bacteria</td></tr>
<tr><td>contig-80_4230_3</td><td>121584</td><td>sugar ABC transporter substrate-binding protein [Paenibacillus sp. VT-400] >gi|835055943|gb|KLU54630.1</td><td>551</td><td>390.963</td><td>390.963</td><td>95</td><td>1.75E-125</td><td>41</td><td>Bacteria</td></tr>
<tr><td>contig-80_650971_1</td><td>121172</td><td>No hit</td><td></td><td></td><td></td><td></td><td></td><td></td><td>No hits</td></tr>
<tr><td>contig-80_29649_6</td><td>119889</td><td>Oligopeptide ABC transporter, periplasmic oligopeptide-binding protein OppA (TC 3.A.1.5.1) [[Clostridium]</td><td>573</td><td>383.645</td><td>383.645</td><td>98</td><td>3.59E-124</td><td>46</td><td>Clostridiaceae</td></tr>
<tr><td>contig-80_3672_3</td><td>117120</td><td>NADH:ubiquinone oxidoreductase [Eubacterium acidaminophilum] >gi|595612498|gb|AHM56143.1| iron</td><td>582</td><td>820.846</td><td>820.846</td><td>99</td><td>0</td><td>66</td><td>Bacteria</td></tr>
<tr><td>contig-80_13664_7</td><td>115796</td><td>ABC transporter substrate-binding protein [Limnochorda pilosa]</td><td>422</td><td>585.874</td><td>585.874</td><td>88</td><td>0</td><td>73</td><td>root</td></tr>
<tr><td>contig-80_91792_2</td><td>113864</td><td>hypothetical protein [[Ruminococcus] torques] >gi|145848012|gb|EDK24930.1| basic membrane protein [</td><td>390</td><td>338.191</td><td>338.191</td><td>99</td><td>2.35E-111</td><td>67</td><td>Clostridiales</td></tr>
<tr><td>contig-80_9933_1</td><td>111172</td><td>formate transporter [Methanoculleus bourgensis] >gi|396938507|emb|CCJ35762.1| formate/nitrite transp</td><td>290</td><td>413.305</td><td>413.305</td><td>99</td><td>1.46E-142</td><td>86</td><td>Methanoculleus</td></tr>
<tr><td>contig-80_18652_2</td><td>109324</td><td>hypothetical protein [Kosmotoga pacifica]</td><td>1364</td><td>1205.66</td><td>2140.482</td><td>96</td><td>0</td><td>57</td><td>Thermotogaceae</td></tr>
<tr><td>contig-80_2215_4</td><td>102178</td><td>hypothetical protein AA931_02620 [Peptococcaceae bacterium 1109]</td><td>706</td><td>954.51</td><td>954.51</td><td>99</td><td>0</td><td>65</td><td>Peptococcaceae</td></tr>
<tr><td>contig-80_40526_3</td><td>101789</td><td>ABC transporter substrate-binding protein [Bacillus sp. FJAT-27997] >gi|899711270|gb|KMY48284.1| ABC</td><td>447</td><td>266.159</td><td>266.159</td><td>95</td><td>3.14E-80</td><td>39</td><td>Bacteria</td></tr>
<tr><td>contig-80_1684_11</td><td>100527</td><td>MULTISPECIES: NADP-dependent alcohol dehydrogenase [Butyrivibrio]</td><td>355</td><td>561.992</td><td>561.992</td><td>98</td><td>0</td><td>75</td><td>Bacteria</td></tr>
<tr><td>contig-80_17330_4</td><td>100441</td><td>ABC transporter substrate-binding protein [Geobacillus sp. C56-T3] >gi|297253216|gb|ADI26662.1| extrac</td><td>556</td><td>399.053</td><td>399.053</td><td>86</td><td>2.74E-128</td><td>44</td><td>Bacteria</td></tr>
<tr><td>contig-80_1410_4</td><td>100129</td><td>hypothetical protein AA931_10905 [Peptococcaceae bacterium 1109]</td><td>818</td><td>1189.48</td><td>1189.48</td><td>99</td><td>0</td><td>68</td><td>Peptococcaceae</td></tr>
<tr><td>contig-80_57905_5</td><td>99665</td><td>methyl-coenzyme M reductase [Methanoculleus bourgensis] >gi|396939406|emb|CCJ36661.1| methyl-coe</td><td>568</td><td>717.227</td><td>717.227</td><td>100</td><td>0</td><td>91</td><td>Archaea</td></tr>
<tr><td>contig-80_3672_2</td><td>99558</td><td>putative PAS/PAC sensor protein [Clostridium sp. CAG:1013]</td><td>557</td><td>592.808</td><td>592.808</td><td>99</td><td>0</td><td>49</td><td>Bacteria</td></tr>
<tr><td>contig-80_110_94</td><td>98888</td><td>methyl-coenzyme M reductase [Methanosarcina sp. E03.2] >gi|931612420|gb|KPL45056.1| methyl-coenzy</td><td>571</td><td>1186.02</td><td>1186.02</td><td>99</td><td>0</td><td>99</td><td>Methanosarcina</td></tr>
<tr><td>contig-80_57603_3</td><td>97671</td><td>hypothetical protein [Thermogemmatispora carboxidivorans]</td><td>412</td><td>206.453</td><td>206.453</td><td>82</td><td>1.47E-57</td><td>32</td><td>Bacteria</td></tr>
<tr><td>contig-80_11012_2</td><td>96689</td><td>LacI family transcriptional regulator [Peptococcaceae bacterium 1109]</td><td>311</td><td>554.673</td><td>554.673</td><td>100</td><td>0</td><td>87</td><td>Peptococcaceae</td></tr>
<tr><td>contig-80_1090054_1</td><td>94753</td><td>hypothetical protein AA931_10905 [Peptococcaceae bacterium 1109]</td><td>818</td><td>104.76</td><td>104.76</td><td>100</td><td>5.34E-24</td><td>66</td><td>Peptococcaceae</td></tr>
<tr><td>contig-80_8113_19</td><td>94574</td><td>translation elongation factor Tu [Treponema lecithinolyticum] >gi|543988696|gb|ERJ91832.1| translation</td><td>395</td><td>727.628</td><td>727.628</td><td>99</td><td>0</td><td>87</td><td>Treponema</td></tr>
<tr><td>contig-80_65396_1</td><td>91677</td><td>hypothetical protein LIP_1185 [Limnochorda pilosa]</td><td>3410</td><td>607.446</td><td>715.6588</td><td>95</td><td>0</td><td>61</td><td>Not assigned</td></tr>
<tr><td>contig-80_373348_1</td><td>91642</td><td>hypothetical protein [Levyella massiliensis]</td><td>457</td><td>293.893</td><td>293.893</td><td>99</td><td>1.19E-93</td><td>66</td><td>Firmicutes</td></tr>
<tr><td>contig-80_2420_8</td><td>91492</td><td>DNA-binding protein [Peptococcaceae bacterium 1109]</td><td>92</td><td>142.895</td><td>142.895</td><td>98</td><td>1.82E-41</td><td>78</td><td>Peptococcaceae</td></tr>
<tr><td>contig-80_15910_7</td><td>91355</td><td>pullulanase [Cellulosilyticum lentocellum] >gi|326542781|gb|ADZ84640.1| pullulanase, type I [Clostridium</td><td>1031</td><td>1055.82</td><td>1055.82</td><td>100</td><td>0</td><td>51</td><td>Firmicutes</td></tr>
<tr><td>contig-80_4865_15</td><td>90114</td><td>peptide-binding protein [Clostridiales bacterium mt11]</td><td>538</td><td>666.766</td><td>666.766</td><td>99</td><td>0</td><td>57</td><td>Clostridiales</td></tr>
<tr><td>contig-80_2106_17</td><td>88125</td><td>sugar ABC transporter substrate-binding protein [Peptococcaceae bacterium 1109]</td><td>527</td><td>804.668</td><td>804.668</td><td>98</td><td>0</td><td>73</td><td>Peptococcaceae</td></tr>
<tr><td>contig-80_52286_4</td><td>86710</td><td>ABC transporter substrate-binding protein [Truepera radiovictrix] >gi|297164729|gb|ADI14440.1| extracel</td><td>501</td><td>384.03</td><td>384.03</td><td>100</td><td>3.68E-126</td><td>51</td><td>Truepera</td></tr>
</table>

Table S6: Results of an iterative protein sequence search using Jackhmmer (hmmer 3.1 package) to identify potential cellulosomal scaffoldin proteins in the metagenomic datasets using a score cutoff of 700

ORF	Cellulosomal scaffoldin query	E-value	score	Assigned to bin ID	
contig-80_10910_2	CipC_[Clostridium_cellulolyticum -	0	2112.4	pb35-1	**Biogas fermenter dataset**
contig-80_24290_1	CipC_[Clostridium_cellulolyticum -	1.2e-221	745.4	pb235-1	
contig-80_7156_9	CipA_[Clostridium_thermocellum] -	0	3156.8	96	
**No Hits**					**Elephant feces dataset**
rumenHiSeq_NODE_1839379_len_402040_cov_3_588501_369	ScaB_[Ruminococcus_flavefaciens_17] -	0	1996		**Cow rumen dataset**
rumenHiSeq_NODE_2071562_len_94063_cov_3_802749_50	ScaB_[Ruminococcus_flavefaciens_17] -	0	1978		
