# Supplementary figures and images for: Deep metagenome and metatranscriptome analyses of microbial communities affiliated with an industrial biogas fermenter, a cow rumen, and elephant feces reveal major differences in carbohydrate hydrolysis strategies
Source: Biotechnol Biofuels. 2016 Jun 7;9:121. doi: 10.1186/s13068-016-0534-x (PMC4897800; doi:10.1186/s13068-016-0534-x)

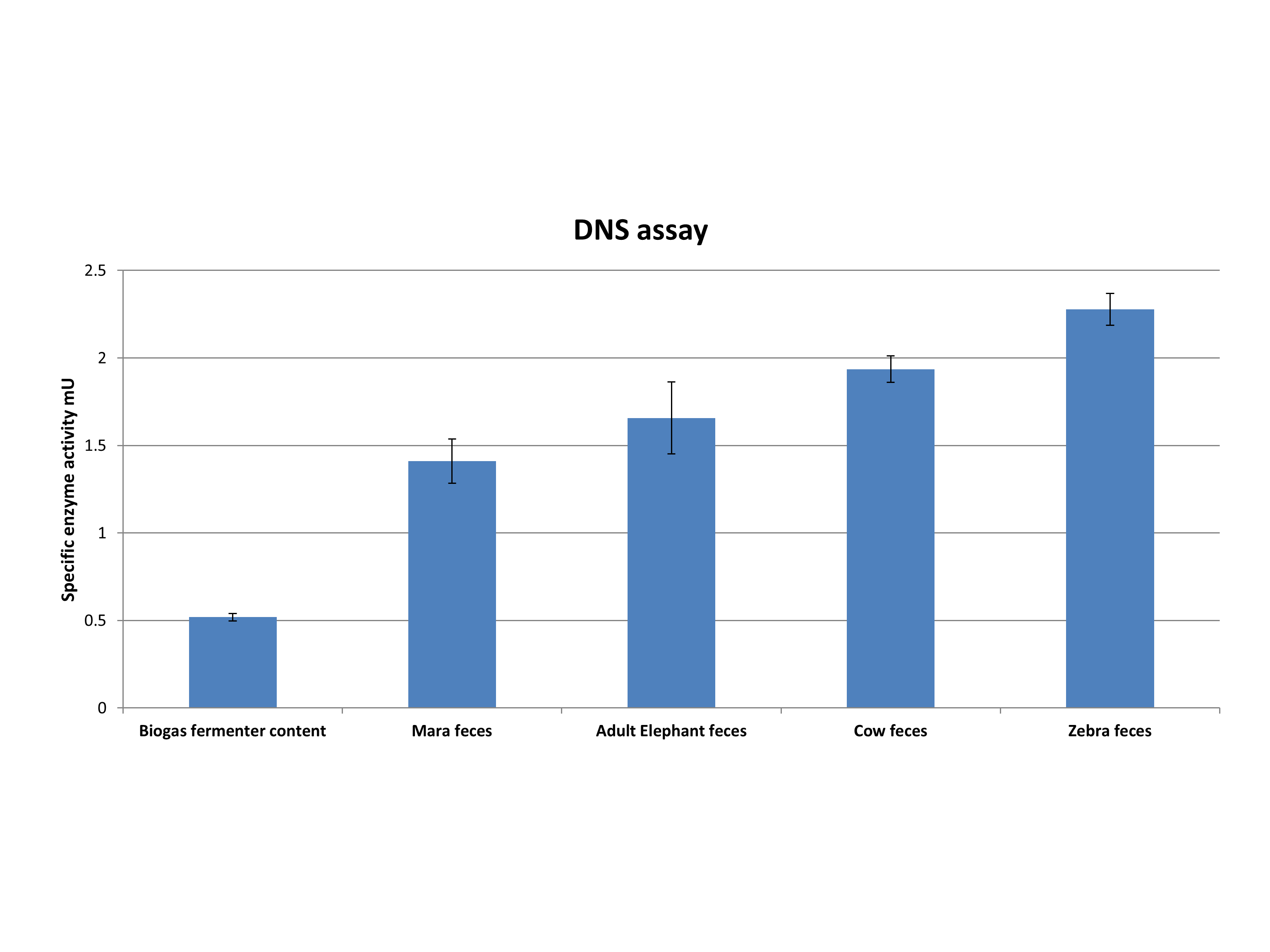

Supplement: Supplementary file 7 — 10.1186/s13068-016-0534-x Comparison of total cellulolytic activities in biogas fermenter content and feces samples of various herbivorous animals determined via 3,5-dinitrosalicylic acid (DNS) assay. [file 13068_2016_534_MOESM7_ESM.png]

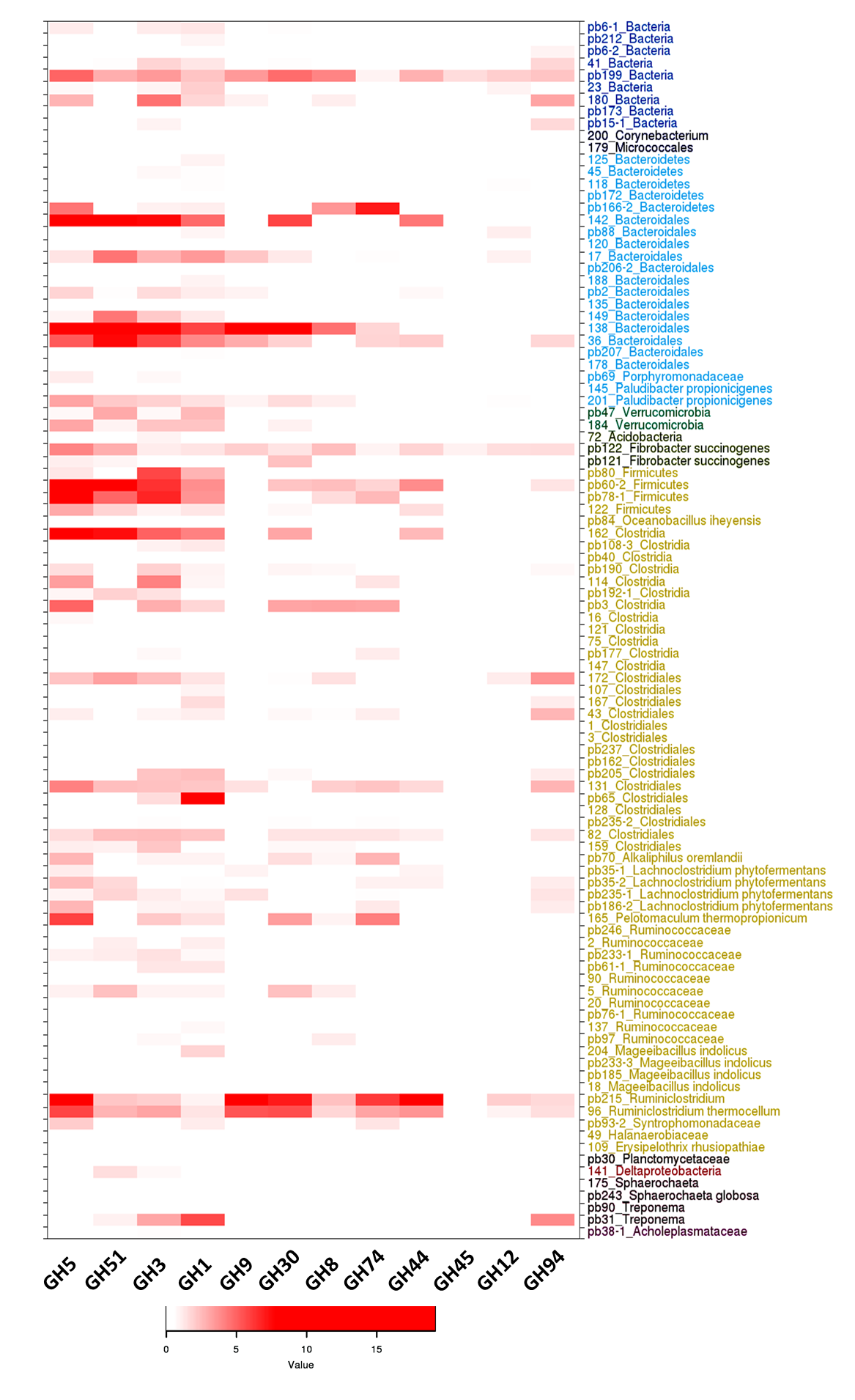

Supplement: Supplementary file 9 — 10.1186/s13068-016-0534-x High-resolution version of Fig. 5a with continuous labeling of all bacterial bins. Bin-IDs are color coded according to assigned phylum. [file 13068_2016_534_MOESM9_ESM.png]
